# Unmet Needs in Systemic Sclerosis Understanding and Treatment: the Knowledge Gaps from a Scientist’s, Clinician’s, and Patient’s Perspective

**DOI:** 10.1007/s12016-017-8636-1

**Published:** 2017-09-02

**Authors:** Marta Cossu, Lorenzo Beretta, Petra Mosterman, Maria J. H. de Hair, Timothy R. D. J. Radstake

**Affiliations:** 1 Department of Rheumatology and Clinical Immunology, University Medical Center Utrecht, Heidelberglaan 100, 3584 CX Utrecht, The Netherlands; 2 Laboratory of Translational Immunology, University Medical Center Utrecht, Utrecht, the Netherlands; 3 Referral Center for Systemic Autoimmune Diseases, Fondazione IRCCS Ca’ Granda Ospedale Maggiore Policlinico, Milan, Italy; 4 Patient Sounding Board of the Department of Rheumatology and Clinical Immunology, University Medical Center Utrecht, Utrecht, the Netherlands

**Keywords:** Systemic sclerosis, Patient-reported outcomes (PROs), Personalized medicine, Clinical unmet needs

## Abstract

Systemic sclerosis (SSc) is a highly heterogeneous disease caused by a complex molecular circuitry. For decades, clinical and molecular research focused on understanding the primary process of fibrosis. More recently, the inflammatory, immunological and vascular components that precede the actual onset of fibrosis, have become a matter of increasing scientific scrutiny. As a consequence, the field has started to realize that the early identification of this syndrome is crucial for optimal clinical care as well as for understanding its pathology. The cause of SSc cannot be appointed to a single molecular pathway but to a multitude of molecular aberrances in a spatial and temporal matter and on the backbone of the patient’s genetic predisposition. These alterations underlie the plethora of signs and symptoms which patients experience and clinicians look for, ultimately culminating in fibrotic features. To solve this complexity, a close interaction among the patient throughout its “journey,” the clinician through its clinical assessments and the researcher with its experimental design, seems to be required. In this review, we aimed to highlight the features of SSc through the eyes of these three professionals, all with their own expertise and opinions. With this unique setup, we underscore the importance of investigating the role of environmental factors in the onset and perpetuation of SSc, of focusing on the earliest signs and symptoms preceding fibrosis and on the application of holistic research approaches that include a multitude of potential molecular alterations in time in an unbiased fashion, in the search for a patient-tailored cure.

## Introduction

Systemic sclerosis (SSc) is a chronic immune-mediated disease characterized by immune system activation, vasculopathy, altered collagen deposition and cellular matrix remodeling culminating in widespread fibrosis. While any organ of the body can virtually be affected by the disease, fibrosis of the skin represents the archetypical feature of SSc, whereas cardiopulmonary complications (interstitial lung disease, ILD; pulmonary arterial hypertension, PAH) currently represent the main cause of morbidity and mortality in SSc patients [1, 2].

An increasing body of the literature is currently focusing on the patients’ disease perception of SSc. These studies clearly demonstrate that physicians and patients have a different perception of the disease [3], as caregivers focus mainly on organ complications and pay less attention to fatigue and pain, which are the major source of complain and distress for patients. Indeed, SSc affects the patients’ quality of life to a higher extent than other chronic autoimmune conditions [4]. In particular, bodily changes not only bear the consequences of progressive disability affecting the different domains of daily life (work and career, family partnering and parenting, loss of social role) but also have deep effects on the perception of self [5]. The loss of self-recognition and the feeling of shame which develop often lead to progressive social isolation. Patients feel poorly understood as SSc is rare, often not known in the general population and sometimes among health professionals as well. The delay in recognizing early symptoms and in the diagnosis, the unpredictability of the different courses of evolution and prognosis and the lack of disease-modifying medications all contribute to an overall perception of great uncertainty and anxiety. SSc is perceived as obscure in its pathogenetic process and in the randomness through which it so heterogeneously affects patients yet falling under the same diagnostic pillar. These topics are also all highlighted in the thematic synthesis of a recent systematic review concerning qualitative studies on patients’ perception in SSc [6].

An empathetic doctor-patient relationship embedded in a regular follow-up structure which ideally begins in the earliest stage of disease and in which communication of information is clear and complete greatly adds to the confidence of patients in the management of care and improves the adherence to treatment. Nevertheless, several qualitative studies all point out a strong demand from patients for a more multidisciplinary, holistic and personalized approach in the SSc field, in which the burden of disease can be better addressed.

Measures such as the implementation of patient-reported outcomes (PROs) could help to establish a more patient-centered approach [7]. However, only a few SSc-specific PROs exist. These are listed in the European League Against Rheumatism (EULAR) Outcomes Measures Library (OML) initiative [8] and have been validated to different extents [9]. Moreover, their proposed use is usually confined to clinical trials and disregarded in daily clinical settings. Domains such as fatigue and anxiety are evaluated only with generic PROs, and others, such as the perception of physical changes and identity or the self-esteem in SSc, are not addressed at all.

As physician-scientists devoted to the care of patients as well as to research, here we want to underline how the abovementioned unmet needs of SSc patients mirror research questions regarding pathogenetic mechanisms, adequate patient classification and treatment possibilities. The struggle of patients in facing the bodily changes developed as end result of the fibrotic process calls for a better understanding of the enigma hidden in the events which drive the progression from early endothelial damage and immune dysregulation to disfiguring and life-threatening fibrotic tissue remodeling. The awareness of patients on the heterogeneity of disease and on the uncertainty about the prognosis highlights the need for a better clinical, molecular and prognostic patient stratification. The lack of satisfaction and trust on the available therapeutic options as well as the demand for a more individualized management of care reflects the strive to develop new targets and treatment strategies among the experts.

In this review, we aim at illustrating the current knowledge on the pathogenic events in SSc, with a focus on early immune and vascular modifications. These alterations bear the potential to drive fibrosis, whose pathways will not be discussed as these have recently been elegantly described [10]. We will rather focus on those events that may help to answer the disturbing and often unanswered questions: “why me?” and “how did it happen?” We will then discuss the current classification approach and the open questions to address in terms of disease monitoring and identification of progressors. Finally, we will provide an overview of the current therapeutic approaches and discuss future options in the light of the concept of personalized medicine. Unique to this review is that we have included the patient perspective in every part of this work, thereby trying to underscore a unique vantage point on what still needs to be done in research and clinical care for patients with SSc.

### A Patient’s View

One always tries to understand ‘How could this happen to me?’ What happened and what caused this avalanche in my immune system? Was it an insect bite? A vaccination perhaps? Didn’t I sleep enough? Or is it just as my husband puts it ‘Your immune system is just quickly distressed.’ For all patients I hope researchers find the cause of all of this some day, so we can try to avoid these stressors.

## Early Pathophysiology in SSc: What Is Happening to the (My) Immune System?

The clinical complexity reflects the multifactorial etiology of SSc. In fact, the most accredited hypothesis on SSc pathogenesis indicates a role for unknown environmental triggers (chemicals, infectious agents) in genetically predisposed individuals to elicit a wide range of epigenetic modifications, conceivably the cause of microvascular damage and immune dysregulation and initiating events which culminate in fibrosis in different patterns and severity degrees [11].

### Environmental Factors, Genetic Susceptibility and Epigenetic Modifications

SSc patients often refer to clinicians their interpretation of the events that led to SSc. Supported by information openly available, they establish links between facts and personal circumstances in a timely manner and propose to their specialist theories that can possibly explain how the disease found its way in their life and ideally identify factors that - once removed - can revert or halt disease evolution. These involve pollutants, infections, diet modifications, occupational factors, stress, and more broadly, all those experiences (big life changes, losses, grief) that are difficult to cope with.

Accordingly, in the literature, there are several reports linking the exposure to different chemicals to the development of SSc and SSc-spectrum symptoms. Silica [12, 13] and solvents [14] (those are mainly linked to an increased risk for men), vinyl chloride [15], gadolinium used as contrast material for magnetic resonance imaging [16], chemotherapeutical bleomycin [17] and pentazocine [18] have all been associated to some extent with an increased risk for SSc or SSc-like syndromes.

Infectious agents such as *Helicobacter pylori* [19] and several viruses have been claimed to play a role in the pathogenesis of the disease, either by molecular mimicry, by providing super antigens -by that triggering immune response and tissue damage— -or by the direct toxic effect on fibroblasts, endothelial cells (EC) and mononuclear cells. In particular, the higher prevalence of Parvovirus B19 in the bone marrow of patients with SSc [20] and the association with increased vascular injury in these patients [21] have been documented. Cytomegalovirus (CMV) and Epstein-Barr virus (EBV) are very prevalent in the general population; the biological likelihood of their role in the pathophysiology of SSc is sustained by their ability to cause persistent infections of monocytes, endothelial cells and fibroblasts, and it is corroborated by association and in vitro studies [22–24]. The composition of the intestinal microbiome as a result of diet, probiotic/antibiotic as well as overall medication intake is being claimed to exert a potentially crucial role in several diseases among which are immune-mediated diseases [25]. The interaction between the microbiome and the immune system is thought to be pivotal for immune homeostasis through direct cell-microbe interaction at the mucosa and indirectly through the release of metabolites which influence a wide variety of biological processes. Therefore, it is logical to hypothesize that also in the pathophysiology of SSc, dysbiosis could be relevant. To date, a very limited number of studies have looked at microbiome in SSc, yet showing with different methodologies (fecal analysis versus mucosal wash and biopsies) consistently overlapping alterations of the intestinal flora [26, 27]. Notably, patients in early stadia (within 2 years from onset) showed the same prevalence of dysbiotic alterations as patients in the late phase, indicating that these might establish before major fibrotic involvement. The association of microbiome modifications with gastrointestinal involvement in SSc does not help to discriminate whether those should be considered cause or consequence for it or to which extent they contribute to disease activity. The field certainly requires further development to ascertain the weight of dysbiosis in SSc pathogenesis.

As for all environmental triggers proposed so far— -to date often referred to as the exposome -the data are intriguing but circumstantial and sometimes conflicting to different degrees. Therefore, hitherto, it has not been possible to establish any true causative relation between the exposome, genetic susceptibility factors and disease onset and/or progression. A major part of this challenge lies in the fact that patients who attend the outpatient clinics have potentially far progressed in the multihit model that led to their disease, making it impossible to understand the series of events, and more importantly, the order of events that might have led to the disease stage they are in when initiating clinical care programs.

To date, the major risk factor for the development of SSc is the presence of a first degree relative with SSc [28]; indeed, SSc heritability is a major concern often leading to anxiety among patients [5]. However, the low concordance for SSc exhibited by homozygotic twins described in the landmark study of Feghali-Bostwick et al. [29] challenges the concept of SSc as a “heritable” condition and rather highlights the relative weight of the epigenetic changes and the need for a broader approach in considering the factors that drive the onset of SSc. Bossini-Castillo et al. have elegantly shown that only 20% of the estimated SSc heritability is to date documented in terms of highly significant and robustly replicated single nucleotide polymorphisms (SNPs) associations [30]. The majority of the SNPs identified resides in the human leukocyte antigen (HLA) region indicating that anomalies in antigen presentations could be important in the pathogenesis of SSc. The other SNPs mainly concern genes involved in the regulation of the immune response [31, 32]. Many of these candidate genes are shared with other autoimmune diseases (AID) though, in particular, the highest overlap (77%) is observed with systemic lupus erythematosus (SLE). Notably, among the numerous *loci* so far identified and replicated, peroxisome proliferator-activated receptor gamma (PPARG, antifibrotic and involved in adipogenesis [33]) and IL-12 receptor beta 1 (IL-12RB1, mediating IL-12-induced Th1 skewing and natural killer [NK] cell activation) are uniquely represented in SSc and not in SLE, rheumatoid arthritis (RA), primary biliary cirrhosis, or celiac disease [30], therefore advocating for preferential functional investigation and targeting in SSc. The functional relevance of these genetic variants in the multihit process leading up to SSc, however, remains enigmatic.

Accumulating evidence emerging from fields outside of the scope of SSc underscores the role of epigenetic changes as a result of the interplay between environmental factors and genetic predisposition, those including DNA methylation, post-translational histone modifications, microRNAs (miRs) and long noncoding RNAs all influencing gene expression without altering DNA sequence.

Among multiple reports for altered DNA methylation in SSc and more specifically affecting fibroblast biology, Wang et al. provided the first direct association between methylation status and immune modifications in SSc [34]. They showed that hypermethylation of the regulatory region of the forkhead box protein 3 (FOXP3) gene -transcription factor crucial for CD4^+^ regulatory T (Treg) cell development -was linked to reduced mRNA expression and circulating Treg cells in SSc and demonstrated that in vitro treatment of CD4^+^ T cells with a methylation inhibitor restored Foxp3 gene expression and Treg development. Regarding the possible role of DNA methylation on vascular injury, hypermethylation of the promoter of the bone morphogenetic protein receptor type-2 (BMPR2) gene has been shown to downregulate the expression of BMPR2 -whose impaired signaling is thought to promote PAH in SSc [35] -on microvascular endothelial cells (MVECs) isolated from affected skin of patients with SSc, therefore exposing them to apoptosis [36]. Histone modifications as well as the expression of a range of miRNAs have been extensively investigated in the context of SSc fibrosis, but hardly in immune cells subsets (reviewed in [11]). Recently, our group showed how miR-618 upregulation in plasmacytoid dendritic cells (pDC) of SSc patients since their prefibrotic stage halts their development and boosts their ability to produce interferon (IFN)-α [37]. The effect of miR-618 on pDCs fits with the presence of type I IFN signature documented in SSc patients since the earliest phase of disease [38] and supports a role for miRs and epigenetic modifications in SSc pathogenesis.

#### A Patient’s View

I think people with SSc are people with an immune system that is easily dysbalanced. During their lifetime they encounter certain triggers that cause a kind of avalanche in the immune system. Sometimes it’s exposed in a form of eczema, the other time your body reacts very fierce on an insect bite, infusion, vaccination, medication or even a facial treatment (the perfume in the day cream gives you red and swollen eyes for example). But also stress takes its toll; not immediately, but after some time (headaches in the weekend after a stressful week). I have always been aware of the sensitiveness of my body. Therefore, I always asked the doctors to reduce the dose of medication and first see how my body responded. They were always surprised by the minimal dose that I needed on several occasions.

I believe, my immune system went on hollow several times, for instance after vaccinations or medication to reduce my cholesterol (preventative). But the doctors always told me that there was no connection between the SSc I now have and that it all happened by chance.

I hope that someday the connection between the type of immune system and the aforementioned triggers are found, to prevent SSc for other people (our children) in the future. And perhaps the sensitiveness of the immune system can become a thing to dose drugs/medication, more closely resembling what is nowadays called personalized medicine.

### The Interplay Between Vascular Damage and Immune Dysregulation in SSc

Endothelial damage expressed as EC activation and apoptosis in response to unknown triggers is considered a primary event in SSc since LeRoy formulated the “vascular hypothesis” in 1975 [39, 40]. In such view, through the years, the focus has been posed on the disturbed biology of endothelial cells (ECs) and on the consequent vascular remodeling resulting in intimal hyperplasia, medial thickening and obliteration of the lumen, loss of capillaries and impaired neoangiogenesis, perivascular inflammation, and prothrombotic state. In particular, endothelial activation markers such as intercellular adhesion molecule (ICAM), vascular cell adhesion molecule (VCAM) and E-selectin are upregulated in the serum and skin of SSc patients [41, 42]. Notably, their expression is increased locally on EC in the early, prominently edematous stage of SSc in respect to the late sclerotic phase, and specifically where mononuclear infiltrates are detectable. Moreover, ICAM and VCAM mediate mononuclear adhesion on dermal fibroblasts in SSc-affected skin, by that further substantiating the link with immune activation and EC damage in SSc [43].

The vascular dysfunction in SSc is expressed also by the lack of balance between vasoconstriction and vasodilation. This phenomenon is illustrated biologically by the upregulation of vasoconstrictive endothelin-1 (ET-1) [44] and altered endothelial nitric oxide (NO) metabolism [45], and it is clinically expressed by Raynaud’s phenomenon (RP) attacks. The consequent repeated ischemia-reperfusion damage results in reactive oxygen species (ROS) release and episodic hypoxia, which becomes chronic as the obliteration of the lumen progresses. Hypoxia and ROS release increase inflammation and tissue destruction and promote fibrotic modifications in the tissues [46].

Vascular endothelial growth factor (VEGF) is a mediator of vascular formation, but it is upregulated in the skin and circulation of SSc patients, where it represents a marker of defective angiogenesis [47, 48]. The chronic upregulation of VEGF in response to hypoxia— -as opposed to acute release -exerts the opposite effect of halting vascular formation. An explanation could be given by the fact that prolonged exposure to VEGF induces the expression of type 1 receptor (VEGFR1) rather than type 2 (VEGFR2), the one actually mediating neoangiogenesis [49]. Alternatively, the preferential expression of antiangiogenic VEGF isoforms in SSc has been documented and could explain the paradoxical effect [50]. VEGF is especially elevated in patients with long disease duration, while circulating endothelial progenitor cells (EPCs) mobilized from the bone marrow (BM) and needed for in situ differentiation into EC appear to be increased in the circulation only in the early stage of fibrotic SSc and not in the late ones [51].

The evidence for vascular dysfunction in SSc relies mainly on studies in definite SSc patients. As described, though, disease duration is a discriminatory factor when looking at the increased local expression of vascular activating factors ICAM, VCAM and E-selectin on EC of the early inflammatory phases, while VEGF, linked to impaired neoangiogenesis, is prominently upregulated in the late fibrotic stage. Valentini et al. reported increased levels of E-selectin already in pre-SSc patients (or early SSc, EaSSc) [52, 53], the ones with a higher risk to develop SSc in comparison to the general population [54, 55]. However, approximately half of the pre-SSc patients they considered had puffy fingers and would be likely now classified as definite SSc patients without fibrotic features, following the new ACR/EULAR classification criteria established in 2013 [56]. In a more recent work from the same group, the new criteria were applied and an increase in the serum levels of ICAM-1 among other circulating chemokines (CXCL8, CCL2) already in the preclinical group in comparison with the healthy control population was reported, with concentrations reaching the highest levels in the definite SSc subsets [57]. VCAM-1 was elevated only in the definite SSc patients group but not in the preclinical subjects. We recently also measured different markers of vascular dysfunction, not only in the EaSSc subsets but also stratifying SSc patients on the basis of the presence or absence of fibrotic features [58]. Our results confirm in a more numerous and strategically stratified cohort the results of Valentini, showing a gradual increase of angiopoietin-2, CXCL16, E-selectin, and ICAM-1 from EaSSc to SSc without fibrotic features and reaching the highest levels in the fibrotic subsets, while levels of VEGF differed from HC only with regard to the fibrotic subsets. These markers of vascular damage should be functionally investigated in the earliest stages of disease, before fibrosis and permanent tissue remodeling occur.

EC activation and adhesion molecule expression allow extravasation of immune cells. Apoptotic ECs in SSc skin were found in early inflammatory stages but not at the latter stage of disease [59], confirming that the endothelial damage is mostly relevant in the initial phase of SSc. Perivascular infiltration of oligoclonal T cells was observed in lesional skin of SSc patients [60], which suggests antigen-driven expansion. The majority of T cells was shown to be CD4^+^; the highest degree of infiltration was observed in early stadia and not only correlated with the extent of fibrotic involvement but predicted fibrotic progression [61]. In contrast, Fuschiotti et al. found that perivascular CD8^+^ T cells were prevalent in early stages; these cells produced pro-fibrotic IL-13 and their presence was suggested to potentially contribute to EC killing [62]. The seemingly contrasting findings could be due to the different methods used for the identification of the T cell subsets and only reinforce the contribution of T cells since the early stage of SSc. Perivascular infiltrates composed of monocytes/macrophages and CD4^+^ T cells in nonlesional skin of SSc patients [63] as well as activated (CD69^+^) T cells and macrophages in affected skin at a higher extent within the first year from SSc onset [64] and of γ/δ T cells prominently in the early, edematous phases [65] are also described. Klein et al. also showed that the percentage of Treg within the CD4^+^ population was diminished when compared to other skin inflammatory diseases [66], while indirect proof of the potential of IL-2-activated CD56^+^ NK in mediating antibody-dependent EC cytotoxicity has been provided [67], corroborating the hypothesis of increased inflammation and poor immune regulation in affected skin in very early fibrotic stages. The skin infiltration could be a direct consequence of the endothelial damage, possibly due to viral infection [68] among the possible triggers, or reflect a primary disturbance in the immune system in SSc.

As in other autoimmune diseases, the type I IFN signature is present in the skin and immune cells of SSc patients. In particular, the expression levels of IFN-induced genes in skin correlate with the severity of skin involvement, as assessed by the modified Rodnan skin score (mRSS) [69]. Most strikingly, we also showed that the type I IFN signature is already present in the circulation of the individuals with increased risk for SSc, the EaSSc subset, and in SSc patients without signs of fibrosis [38]. Noteworthy, in these earliest phases of disease -and in the nonfibrotic patients to the greatest extent -the highest prevalence of type I IFN signature and the highest averages of IFN scores were observed. Type I IFN signature is common in many autoimmune conditions, in SLE in particular [70]), but in contrast to SLE, in SSc, this signature seems to mark more potently the early phases of disease characterized by the absence of fibrosis, suggesting an early activation of the pDC compartment and possibly driving all further molecular events leading to the establishment of what we call SSc and its progression.

The presence of IFN signature directly inflicts Toll-like receptor (TLR)-mediated activation via immune complexes formed by autoantibodies and/or an exaggerated response to virus triggers or endogenous ligands. There is accumulating evidence supporting a hyperactivated state of pDC and of myeloid cells in SSc, as well as an enhanced response to TLR-mediated stimuli. For instance, CXCL4, a biomarker correlating with the presence and progression of skin fibrosis, ILD, and PAH in SSc, seems preferentially and spontaneously released by pDC [71]. The monocyte/macrophage lineage in both its M1 (classical activated, proinflammatory) and M2 (alternatively activated, tissue-remodeling and pro-fibrotic) components is considered to contribute to the pathology of SSc in different fashions, likely reflecting the heterogeneity of the disease over time (summarized in [72]). Furthermore, SSc monocytes and dendritic cells (DC)— -and more specifically myeloid (mDC) and monocyte-derived (moDC) subsets— -have shown augmented response to different TLR stimuli when compared to healthy donors [73], and interestingly, the cytokine production was clearly distinct when comparing the early (< 2 years from onset) and late disease states and throughout the different SSc subsets, again highlighting the complexity of SSc and the presence of diverse biological processes being at play during the different disease states. TLR ligands are expressed by several pathogens and are also derived in inflammatory processes as a result of tissue damage/cell death; also in SSc, the presence of circulating endogenous TLR4 ligands has been shown [74]. TLR-mediated hyperactivation and release of type I IFN and proinflammatory cytokines could itself trigger a break of tolerance in predisposed individuals [75] promoting the activation of autoreactive T cell clones.

T cell polarization toward Th2 and Th17 has been documented in SSc and linked to the fibrotic process. The role of Th17 cells, though, is very controversial and in humans could reflect more the inflammatory phase than being causative in fibrosis. In fact, although IL-17 and Th17 cells have been found to be increased in the circulation and in the skin of SSc patients -most intriguingly in the early stage of disease -to date, there is no clear evidence showing the direct role of Th17 cells in fibrosis [76, 77]. Th17 cell supernatants rather seem to trigger a proinflammatory phenotype both in healthy and SSc dermal fibroblasts [78], supporting a proinflammatory role for Th17 in the initial stadia of SSc. The profibrotic role carried by Th2 cells is far more established and finds evidence for CD4^+^ [79], CD8^+^ [62], and CD4^+^CD8^+^ [80] T cell activation in the circulation and affected tissues of SSc patients. The Th2 “supremacy” could represent a form of excessive attempt to repair endothelial damage, as Th2 expansion is well documented in wound-healing processes [81]. Nevertheless, a Th1 skewing -as suggested by the involvement of the abovementioned IL-12 pathway -cannot be excluded in SSc [82] and could be a signature of the latest phases during fibrosis resolution. Alongside the clues toward a hyperactivation of the immune system, a defect in the feedback mechanisms limiting the magnitude of the immune response has being addressed. In particular, T regulatory cells [83] and CD56^+^ NK cells [84] have been shown to exert an impaired function in SSc. In NK cells, the defective cytotoxicity is described in the fibrotic form of disease. Recently, we have shown that EaSSc individuals and patients with definite SSc without fibrosis show an enhanced response of NK and NKT-like CD56^+^ cells to TLR stimuli [85], which rather points toward a state of hyperactivation for both cell populations. It would be of great interest to assess the cytotoxicity capability in the early, prefibrotic stages and the TLR response in the late, fibrotic phase, to gain more insights into the function of NK and NKT-like cells in a timely manner. The proinflammatory phenotype exhibited in the early disease could lead the same cell populations to exhaustion in a chronic activation setting and contribute to impaired immune regulation in more advanced, fibrotic SSc. CD56^+^CD3^+^ NKT-like cells have hardly been studied in the context of SSc -where their number has been shown to be reduced in the circulation of definite SSc patients, but not EaSSc individuals [86]— -and deserve further research in the field.

A great debate has developed over the question whether SSc should be considered an autoimmune disease, given the presence of specific autoantibodies even before the clinical onset and their accuracy in predicting disease phenotype and prognosis (discussed in the next paragraph of this review). A pathogenic role has been claimed for some of them, as in the case of anti-endothelial cell antibodies [87], where EC apoptosis would indicate a direct cell damage through specific antigen recognition. The potential of immune complexes formed by anti-topoisomerase I antibodies bound to nuclear extracts in the serum of SSc patients has been explored by Kim et al., showing their capacity to enhance type I IFN response in pDC [88], by that amplifying the inflammatory loop in SSc. Certainly, the presence of autoantibodies reflects an activation of the B cell compartment in SSc, but their role in initiating or maintaining pathogenetic pathways requires further exploration.

In conclusion, a substantial body of evidence that has accumulated over the years suggests the presence of a complex spatial and time-dependent, multilayered molecular system starting with vascular damage somehow leading to ongoing inflammation and culminating in fibrosis. Hitherto, most of the research aimed at unraveling this complex system focuses on single molecules and/or pathways of interest. On the contrary, evidence is building that such focused approach is not going to lead to a full understanding of disease process and suggests that a more holistic approach is needed to do so. For instance, more recent papers show that the power of omic wide analysis of the transcriptome [89], proteome [90], methylome or combinations of these [91, 92] in cellular subsets rather than whole blood provides paradigm shifts in our understanding of cellular biology. These techniques are in its infancy in terms of being applied to decipher human diseases but will show their relevance in improving disease understanding within the coming years.

#### A Patient’s View

When I read all the results of years of study and hard work of doctors, researchers and all those involved, one just can only have deep respect. It’s a complex matter, that’s for sure.

For me as a patient it is a complex disease to describe to a doctor for several reasons.

At first, an immunological disease presents itself in many ways, and dependent on the form of that moment you are referred to a different doctor. Or the doctor picks out one thing and leaves the rest for the moment, but maybe that might actually be the reason to attend the doctor. Most of the time you experience your body in an unwillingly way, you leave it be, take some rest and often it will go away, or not.

Secondly, dependent on the knowledge of the specialist you meet, there is another part of the puzzle unrevealed, or not. But now I see that each intervention could have triggered some aspects of the malfunctioning of my body and I can also understand that even when you present the right complaint to the right doctor, and in a proper way so he/she can link it to -at least a piece of -the disease, still the knowledge and the expertise of the physician are crucial for a correct diagnosis, to refer the patient to a center with expertise for SSc.

Lastly, I take good care of my body, always did; eat healthy, sport enough (but that’s easy, because I like that), take complaints seriously by going to a doctor when the complaints don’t vanish by themselves. Is SSc iatrogenic? Shouldn’t I had been so persistent on a good diagnosis, because I always knew it couldn’t be just rheumatoid arthritis? A high level of cholesterol by familial hypercholesterolemia? Did I make it worse myself?

I am left with all these questions. What impact has stress in childhood or puberty or childbirth etcetera in this play of the cells of the immune system? How many diseases can we connect to just one overall dysfunctioning immune system?

## Classification of Disease: Who to Fit Where

### Raynaud’s Phenomenon and the Concept of “Early SSc”

The clinical spectrum and prognosis of SSc are highly heterogeneous, but strikingly in at least 95% of the cases, the onset of disease is preceded -sometimes by years -by the occurrence of Raynaud’s phenomenon (RP) [93]. RP reflects an impaired balance between vasoconstriction and vasodilation in response to different stimuli -mainly cold or emotional stress -and it is characterized by episodic color changes of the extremities (predominantly the digits) turning white (ischemia), blue (cyanosis), and red (reperfusion). RP is common also in the general population and is most often harmless (primary, PRP) [94], but in a small percentage of cases, RP is the alarm bell for an underlying disease. In several autoimmune conditions, RP is a presenting sign or a complication of a long-lasting disease, including SLE, RA, Sjogren’s syndrome (SjS) or other connective tissue diseases in general. However, it is SSc that accounts for most of the cases of the so-called secondary RP whose occurrence in a previously healthy individual should always raise the suspicion of a developing sclerodermatous condition. This, of course, naturally leads to a key question: is it possible to identify the source of RP and early identify the presence of an underlying pathological conditions? Several lines of evidence indicate that in PRP, vascular abnormalities are mostly functional, while in secondary RP, especially in SSc-related RP, structural endothelial alterations can be observed [95]. Nailfold videocapillaroscopy (NVC) has slowly emerged as a necessary tool to visually highlight microvascular alterations and the endothelial derangement which characterize SSc. The NVC patterns of SSc-related microangiopathy are now well characterized and formalized from a qualitative and quantitative point of view [96].

With the recognition of vasculopathy as a pivotal sign of SSc along with the presence of typical laboratory markers of autoimmune system activation, LeRoy and Medsger proposed a set of criteria to characterize a secondary RP bearing the prototypical characteristics of SSc [52]. In these criteria, the combination of RP with the presence of SSc-specific antinuclear autoantibodies (ANA) -such as anti-topoisomerase I (ATA), anti-centromere (ACA) and anti-RNA polymerase III (RNAPIII) -and SSc-specific NVC changes was sufficient to define a clinical entity which was labeled as “the most limited form of SSc”, lSSc (limited SSc) or early SSc (hereto referred as “EaSSc”). These criteria were controversial and raised the provocative question “when is scleroderma really scleroderma?” as it was doubtful whether subjects without other symptoms or sclerodermatous characteristics were really likely to progress toward a clinically manifest SSc [97]. The landmark study from Koenig et al. [54] mostly addressed this issue. In a 20-year prospective study of 586 RP patients, they confirmed SSc-specific autoantibodies and NVC modifications as independent predictors of SSc (adjusted hazard ratios for ANA positivity, SSc-specific autoantibodies, and NVC changes, respectively, 5.67, 4.7, and 4.5) [54]. In particular, the presence of both specific autoantibodies and NVC changes conferred a 60-times increased risk to develop SSc when compared to patients with RP without these features, with progression rates to definite SSc of 47% at 5 years, 69% at 10 years and 79% at 15 years. Intriguingly, these authors concluded that “given enough time most early SSc patients will develop definite SSc”. These findings are of paramount importance as the early recognition of this preclinical subset may constitute a “window of opportunity” to finally answer to the “why me?” questions formulated by patients or to provide some form of prognostication eagerly awaited by patients and relatives. It is now recognized that EaSSc patients are not a homogenous group and that different subjects progress toward a definite SSc with different rates, sometimes earlier and sometimes later, in some cases toward a severe and aggressive disease, and in other cases toward a smoldering condition. The study of EaSSc may thus solve several questions related to the factors are that are associated with faster or worse evolutions and to the understanding of the molecular pathways that lead to organ damage.

Since the work of Koenig et al., many studies tried to provide an optimal characterization of EaSSc patients and of the factors associated with disease progression. Ingegnoli et al. confirmed that NVC alterations and that the occurrence of SSc-specific autoantibodies are predictors of progression from RP to SSc after 5 years from the initial evaluation; the authors also proposed a predictive model based on these alterations with good internal prognostic discrimination accuracy, albeit a thorough internal or external validation was not made [98]. The importance of NVC and autoantibody determination in the transition from EaSSc to a definite SSc was also confirmed by two other independent Italian groups [55, 99]. In [99], as in Koenig et al., it was observed that the risk of progression toward a definite SSc was higher for those patients with both NVC alterations and SSc-specific autoantibodies, while the risk was markedly reduced in the absence of immunological alterations. Vigone et al. also showed that a more severe capillaroscopic pattern is associated with higher rates of progression and faster evolution times to definite SSc; similarly, patients with or without ACA had different evolution rates, with ACA associated with a slower evolution over time (median evolution time 55 versus 23 months for ACA-positive versus ACA-negative patients). In the same work, the DQ5-DR1 haplotype strongly reduces the risk of progression and lengthens the time to evolution independently of the presence of ACAs. These results strengthen the notion that factors influencing and linked to immune activation, as also reviewed in the pathogenesis chapter, may have a role in disease progression.

### SSc Classification: a Paradigm Shift from Old to New Criteria

Since the publication of the 1980 SSc classification criteria, it was recognized that these lacked enough sensitivity to recognize patients with early disease, especially with no or limited skin involvement [100]. After the publication of these criteria, the importance of endothelial alterations in the pathogenesis and progression of SSc has been steadily recognized as suggested by the relevance of NVC findings in this context. Similarly, it was observed that the 1980 criteria did not give any weight or importance to the determination of autoantibodies, whose patterns are indeed relevant to disease progression or to determine the risk of internal organ involvement [101]. Attempts to incorporate NVC and autoantibody findings as well as other early features of SSc in the preexisting classification criteria proved effective in increasing their sensitivity and specificity [102]. In 2013, the European League Against Rheumatism (EULAR) and the ACR joint endeavor produced a new set of classification criteria capable of identifying with higher sensitivity patients with limited or no cutaneous sclerotic features. In the 1980 criteria for the classification of SSc, fibrosis was the anchor sign of SSc, and to be classified, a patient had to show typical sclerodermatous changes proximally to metacarpophalangeal or metatarsophalangeal joints (major criterion) or, alternatively, present with at least two between sclerodactyly, pitting scars/digital ulcers, and bibasilar lung fibrosis (minor criteria) [100]. The 2013 ACR/EULAR criteria include a higher number of features (vascular, immunologic, fibrotic) which determine the classification on the basis of a weighted score that each feature bears; a score ≥ 9 is sufficient for SSc classification [56]. In this new system, the major criterion of the 1980 classification is still valid and sufficient for SSc classification, because alone it accounts for 9 points. RP, SSc-specific autoantibodies and SSc-specific NVC features which define EaSSc account for 8 points (3, 3, and 2 points, respectively). The occurrence of any extra scored item on top of the 8 points (EaSSc score) automatically classifies the patient as SSc [103]. An overview of the different features considered as classification criteria in the different systems adopted through time is provided in Table 1.

**Table 1:** Comparison of different classification criteria considered in different classification systems

	1980 SSc (ARA)	2013 SSc (ACR/EULAR)
RP	N	Y
NVC (SSc patterns)	N	Y
Autoantibodies	N	Y
ANA (aspecific)	na	N
ATA	na	Y
ACA	na	Y
RNAPIII	na	Y
Skin involvement		
Sclerodermatous, proximal to MCP	Y (major)	Y (suff)
Sclerodermatous, distal to MCP		
Sclerodactyly	Y (minor)	Y
PF	N	Y
Fingertip lesions		
DU	Y (minor)	Y
PS	Y (minor)	Y
Telangiectasia	N	Y
Internal organs involvement		
ILD	Y (minor)	Y
PAH	N	Y

*1980 SSc (ARA)* 1980 Preliminary criteria for the classification of systemic sclerosis (SSc) [100], *2013 SSc (ACR/EULAR)* 2013 American College of Rheumatology/European League Against Rheumatism classification criteria for systemic sclerosis [56], *RP* Raynaud’s phenomenon, *NVC* nailfold videocapillaroscopy, *ANA* antinuclear autoantibodies, *ATA* anti-topoisomerase I antibodies, *ACA* anti-centromere autoantibodies, *RNAPIII* anti-RNA polymerase III autoantibodies, *MCP* metacarpophalangeal joint, *PF* puffy finger, *DU* digital ulcer, *PS* pitting scar, *ILD* interstitial lung disease, *PAH* pulmonary arterial hypertension, *N* not considered as criteria, *na* not applicable, *Y* considered as criteria. *suff* sufficient criteria for classification

The main novelty of the new classification system resides in the fact that other nonfibrotic features of SSc such as puffy fingers (PF), telangiectasia, and digital ulcers are actually among the weighted items and, when in combination with the triad that defines EaSSc patients, are sufficient to classify an individual as definite SSc patient. Thus, the application of these criteria may allow the identification of those subjects without skin involvement or, most importantly, from a prospective and temporal point of view, of those patients with prefibrotic features (PF). The 2013 ACR/EULAR classification criteria can identify patients with early disease (< 2 years from onset) and the ones with lcSSc subset with high sensitivity [104], which allows the inclusion in clinical trials and research studies also of patients with milder disease.

From a biological and clinical perspective, patients with EaSSc and definite SSc without fibrotic features and patients with limited (lcSSc) and diffuse (dcSSc) cutaneous involvement have well distinct characteristics [58]. A number of vascular dysfunction markers show a linear increasing trend along these subsets. Similarly, other clinical and laboratory parameters, such as the erythrocyte sedimentation rate, C-reactive protein levels, hypergammaglobulinemia, diffusing capacity for carbon monoxide, and forced vital capacity, worsen from EaSSc to definite SSc to the subsets with overt fibrosis.

### The Challenge of Identifying Progressors

The identification of factors that may help to identify patients at higher risk of progression from EaSSc to definite SSc is a young field of research and many discoveries are still to come. Hopefully, these discoveries will allow early prognostication and disease interception, albeit it is still doubtful how and when to treat subjects with EaSSc and to what extent the current therapies may slow the evolution of SSc. Besides these considerations, the diagnosis of EaSSc remains challenging, due to the poor awareness about the potential consequences of RP in the general population, by general practitioners or the difficulties in accessing NVC or autoantibody testing and/or the costs of these exams for low-income people. Prognostication still remains a fundamental exercise in every SSc patients, including those with a definite diagnosis, overt fibrosis and a long-lasting disease. The prognosis in SSc patients with severe skin and internal organs involvement is poor, as the 9-year cumulative survival does not reach 40%, whereas in patients with milder clinical phenotypes, the 9-year cumulative survival can attain 80% [105]. Therefore, a thorough stratification of SSc patients is crucial for an adequate medical follow-up; moreover, as pointed out in [106], “both patients and their families may later regret being over-optimistic about their prognosis” and “patients are willing to have access to accurate prognostic information.” At the best of the current knowledge, the scientific community is trying to identify the different stages in SSc, with the aim to stop, reverse or prevent the fibrotic process, the major cause of morbidity and disability in SSc.

As already stated, autoantibody specificity is fundamental to guide the clinical follow-up and monitor more closely for diffuse cutaneous involvement (Scl-70ATA, RNAPIII specificities), ILD (anti-topoisomerase I), SSc renal crisis (RNAPIII), and PAH (ACA) [101]. There is a growing literature to promote the use of NVC not only in the diagnostic stage but to monitor the changes of the patterns through time in correlation with the progression of disease and the development of specific features. Several morphologic nailfold changes mirroring different aspects of microangiopathy in SSc have been described in combined patterns (“early,” “active” and “late”) [96, 107]. These patterns are specific for SSc and are defined as “scleroderma pattern”. They sequentially document not only the progression of vascular damage through time but also the development of clinical complications [54, 108]. In particular, among the patients who evolve from the “early” to the “active” and finally to the “late” pattern, the prevalence of internal organ involvement is higher and there is correlation with disease severity, when compared to patients with slower capillaroscopic turnover who for a longer time exhibit the “early” pattern and whose clinical evolution is milder [109, 110]. Specific NVC modifications and patterns have been associated with the development of distinct SSc features (reviewed in [111]) and have shown to predict organ involvement, more specifically of ILD, with increasing risk from “early” to “active” to “late” pattern [112]. The gradually increasing predictive value from “early” to “active” to “late” pattern was later attested for all nine organ systems [110] according to the Medsger disease severity scale [113]. Currently, the use of capillaroscopy and of a prognostic scoring system to predict DU development at 6 months has been ascertained and validated [114, 115], as determined by scoring the magnitude of capillary loss (qualitatively described in the “late” pattern) combined with the presence or the absence of digital ulcers and critical ischemia at baseline. The implementation of videocapillaroscopy in clinical practice seems critical for an accurate follow-up.

It is widely accepted to classify SSc patients into different subsets based on the extent and localization of the prototypical skin fibrotic changes. In particular, patients affected only distally to elbows, knees, and clavicles are referred to as having the limited cutaneous (lcSSc) form and patients presenting with proximal fibrosis are defined as having diffuse cutaneous (dcSSc) SSc [116]. dcSSc patients have the shortest time to first non-Raynaud symptom and the fastest rate of development of complications [117]. dcSSc also presents more often with clear signs of systemic inflammation, with increased circulating C-reactive protein being itself associated with poor survival and shorter disease duration in dcSSc [118], as opposed to lcSSc, where the course of disease is generally milder with smoldering progression of fibrosis but still high chance to develop critical disability and severe organ involvement in the long run [119]. The separation into lcSSc and dcSSc helps the clinician to plan diagnostic assessments and estimate the risk of developing complications, by that improving the quality of follow-up and surveillance. In particular, a prediction rule for the development of the most severe dcSSc subset on the basis of gender, time of first non-RP symptom, puffy hands/sclerodactyly, and autoantibodies specificity has recently been proposed [120]; with a sensitivity of 87% in recognizing dcSSc cases and a specificity of 61% in excluding lcSSc cases, the accuracy is not optimal but surely provides an easily applicable screening method for the initial risk stratification of patients. Medical modeling has also been applied to early dcSSc to find the predictor of mortality based on clinical parameters and simple laboratory tests, and internal and external validation showed that these models can be applied to different populations with promising results [121, 122].

Nevertheless, the subsetting into lcSSc and dcSSc does not fully account for the clinical heterogeneity and the unpredictable, diverse response to therapy which characterizes SSc. With the increasing knowledge about the molecular mechanisms underlying SSc pathogenesis and the reduction in cost of molecular biology techniques, a great emphasis is currently placed on the discovery of biomarkers and on the reclassification of diseases based on transcriptome, epigenome, genome, cytokine and metabolome information [123], and SSc is no exception. Using information from skin biopsies and gene transcript, US researchers identified specific signatures associated with the severity of interstitial lung involvement [124] or with the extent of skin fibrosis [125]. Intriguingly, in the latter report, it was observed that a specific signature could be used to distinguish treatment responders from nonresponders prior to change in skin fibrosis. While these studies clearly demonstrate that a reclassification of SSc based on biomarkers is feasible and could be used to stratify patients, the true value of a molecular reclassification of SSc must be further examined. The complexity of the disease, and maybe SSc in particular, may require a more holistic approach to identify novel mechanistic biomarkers. Such holistic— -otherwise called omic-wide techniques— -have recently been shown to yield novel data with high impact [92]. Such untargeted approach has been applied in SSc before in terms of proteomics on pDCs from different stages of SSc by which CXCL4 was identified [71]. In the last years, CXCL4 has emerged as a potential candidate in this setting as increased plasma levels of CXCL4 can be observed in EaSSc patients as well as in fibrotic subjects with plasma concentrations correlating and correlate with disease severity [71]. Moreover, CXCL4 concentrations are affected by therapy [126] and a model that incorporates CXCL4 has been proposed to stratify responses to imatinib [127].

An adequate redefinition of SSc subset based on functional classification, that is on factors that account for disease severity and progression, obviously requires an adequate definition of disease severity and progression. To date, there is no consensus on how to define progression in SSc, and activity indexes are still preliminary and require extensive validation before they can be used on a wide SSc population encompassing also preclinical subjects [128]. Recent endeavors have provided new tools to assess pharmacological responses in clinical trials [129]; the newly developed composite response index in dcSSc (CRISS) includes core items that assess change in skin and lung domains, disability and physician and patient global assessments. Despite the merits and the novelty of this index that encompasses several aspects of the disease, its application in a wide SSc population that includes non-early dcSSc or lcSSc cases is controversial. Moreover, as the same authors acknowledge, no input was sought from patients during the construction of this index. Hence, while the effect of drugs on several physical and functional aspects of SSc is likely to be captured by the CRISS index, the efficacy of the same drugs from the patient’s perspective is difficult to be ascertained. In general, these aspects have mostly been neglected, and while several trials have used some self-reported measures as secondary endpoints to measure treatment efficacy, such as the short form (SF-36) health survey or the Euro quol questionnaire (EQ-5D), more focused and structured PROs have seldom been used in SSc. Recently, the Self-Efficacy for Managing Chronic Disease (SEMCD) scale has been validated in SSc [130] and indeed, it will be of interest to compare therapy responses by scales created by the physician’s judgment with those indexes that take into account fatigue, physical discomfort and pain, emotional distress, interference of health problems in daily life activities and independence. More specific PROs that assess specific domains (gastrointestinal, circulatory to name a few) have been validated and can be used to gauge disease progression and response to therapy from the patient’s perspective [9]; however, more specific instruments to capture the complexity of the disease or that combine clinical and patient-based endpoints are needed.

#### A Patient’s View

Disease criteria are important to diagnose, preferably at an early stage, or even predict the course of the disease if not intervened. I think that it’s always hard for a physician to decide to let a disease take its course whilst there is great uncertainty about its outcome. For instance, almost every time I saw a new rheumatologist (people move houses, retire, change jobs, etc.), they wanted me to get off my medication. At first, I was cooperative and reduced medication, which always ended up in a flare of the disease and me being a year crippled, not really fit for work and a household that couldn’t fully count on me, because I couldn’t walk or do something else. Because of my family and my personal inconvenience, I decided not to be so cooperative anymore in these experiments. But what other ways are there for a doctor in ‘knowing the patient’, experience the effects of interventions and so? Every patient is different. Disease criteria are a very important answer to this I think, as it can help even the more unexperienced doctor to know what to do at what time in the patient’s journey.

On the other hand, such criteria shouldn’t be followed as a rule, they should be fit for each patient such as true personalized medicine is truly meant.

## Evidence-Based Approach to the Treatment of SSc and the Need for Personalized Medicine

SSc is a highly heterogeneous disease. Differences in clinical presentation among patients as well as the variable degree of organ involvement that patients may experience make challenging the treatment of the disease and of its complications. Currently, there is no single drug to treat SSc, and the pharmacological approach is based on a combination of drugs that may be effective in treating organ damage or in relieving symptoms associated with visceral involvement. An individualized approach is often required to optimize the treatment of SSc and the patient’s response yet with often unpredictable responses.

In 2009, the EULAR issued a set of recommendations for the treatment of SSc-related organ complications [131]. Overall, 14 recommendations were produced as a result of literature review and of expert consensus, covering the following aspects of the disease: Raynaud’s phenomenon (RP), digital ulcers (DUs), pulmonary arterial hypertension (PAH), skin and lung disease, scleroderma renal crisis (SRC) and GI involvement. At the end of 2016, these recommendations were updated including in the expert panel the two patients nominated by the pan-European patient association for SSc (Federation of European Scleroderma Associations [FESCA]) (Table 2) [132]. The EULAR recommendations are the result of a huge process of literature review: 8771 papers were considered (5436 already considered for the 2009 recommendations and 3335 for the updated recommendations); of these, 462 were deeply analyzed (281 already considered for the 2009 recommendations and 181 for the updated recommendations). Despite this effort, no indications and data were produced regarding immunosuppressive and experimental therapies for which no published or complete data existed at the time of literature review.

**Table 2:** EULAR recommendations for treatment of systemic sclerosis

Domain	Recommendation	Literature evidence
Raynaud’s phenomenon	Dihydropyridine calcium antagonists (i.e., oral nifedipine) should be considered as first-line therapy for Raynaud’s phenomenon.	Two RCT
	PDE-5 inhibitors should also be considered in the treatment of Raynaud’s phenomenon.	Literature meta-analysis
	Intravenous iloprost should be considered for severe Raynaud’s phenomenon after failure of oral therapy according to experts’ opinion.	Literature meta-analysis
	Fluoxetine might be considered in the treatment of SSc-RP attacks.	Minor evidence from a small study
Digital ulcers	Intravenous iloprost should be considered in the treatment of digital ulcers in patients with SSc.	Results from 2 RCT
	PDE-5 inhibitors should be considered in the treatment of digital ulcers in patients with SSc.	Results from 1 RCT and meta-analysis In one small RCT, PDE-5 inhibitors may prevent the development of new digital ulcers.
	Bosentan should be considered for the reduction (prevention) of new digital ulcers in SSc, especially in patients with multiple digital ulcers despite use of calcium channel blockers, PDE-5 inhibitors, or iloprost therapy.	Results from 2 large RCT
Pulmonary hypertensions	ERA, PDE-5 inhibitors, or riociguat should be considered to treat SSc-related PAH.	RCT in PAH patients that include PHA secondary to CTD
	Intravenous epoprostenol should be considered for the treatment of patients with severe SSc-PAH (classes III and IV).	RCT with mixed PAH population
	Prostacyclin analogues (inhalatory iloprost; subcutaneous treprostinil) should be considered for the treatment of patients with SSc-PAH.	RCTs
Skin and lung	Methotrexate may be considered for the treatment of skin manifestations of early diffuse SSc; no data are available about the effect on lung function.	Two RCTs
	Cyclophosphamide should be considered for the treatment of interstitial lung disease (especially if progressive).	One RCT
	HSCT should be considered for the treatment of selected patients with rapidly progressive SSc at risk of organ failure. Careful patient selection is mandatory due to high risk of treatment-related side effects and of early treatment-related mortality.	Two RCT comparing HSCT to cyclophosphamide
Scleroderma renal crisis	ACE inhibitors should immediately be used in the treatment of scleroderma renal crisis.	Review of survival data and several cohort studies
	Glucocorticoids should be carefully used in patients at risk for scleroderma renal crisis; blood pressure and renal function monitoring is required.	Retrospective data
Gastrointestinal	PPI should be used to prevent esophagitis.	None
	Prokinetics should be used for the management of SSc-related symptomatic motility disturbances.	Limited, small studies
	Rotation antibiotics should be used to treat symptomatic small intestine bacterial overgrowth.	Limited, small studies

Modified from [132]

*RCT* randomized controlled trials, *PDE* phosphodiesterase, *CTD* connective tissue diseases, *HSCT* hematopoietic stem cell transplantation

Currently, several trials are ongoing to evaluate the possible effect of synthetic conventional drugs, mainly immunosuppressive therapies or newer drugs that specifically target potential pathways of interest in SSc pathogenesis. The potential candidate molecular therapies for the different aspects of SSc are discussed in [133] and include therapies to restore endothelial homeostasis and to treat vasculopathy, to tackle inflammation and immune system activation, or to treat fibrosis and aberrant collagen production and deposition.

Whatever the treatment and the statistical significance reached by primary endpoints of clinical trials, the overall responses are far from being dramatic and several patients exposed in the real world to approved drugs have little or no benefit from them. There are many reasons to explain disappointing results of otherwise promising drugs in systemic sclerosis. As already discussed in the classification chapter, there is no clear consensus on how to define disease severity, progression, activity, and in general, on what are the best tools to assess pharmacological responses in a wide SSc population. Secondly, patient selection in clinical trials may not reflect the “true” overall SSc population and often, results obtained in a particular subset of patients, as for instance early dcSSc, are translated to other subjects without clear evidence for a potential benefit. Thirdly, statistical significance may not always coincide with a meaningful clinical difference; while for some clinical domains the concept of “minimally clinical important difference” is well-established [134–138], this is not true for many aspects of SSc-related complications and not always these are taken into account in designing clinical trials. Even worse, and almost inevitably, these differences are learned after a single pivotal trial has been concluded [134, 136], leading to a waste of valuable information even if the necessary groundwork for future trials is posed. Lastly, post hoc analysis to identify the factors that cause some of the people in a trial to be responsive is rarely performed. Hence, current available guidelines and recommendations do not provide any insight about patients’ stratification to optimize therapy outcomes and responses.

A major source of uncertainty in individual responses to therapy resides in the way the trials themselves are designed and conducted. Canonical trials involve a large number of subjects, hopefully hundreds of people representative of the general population of interest, which are exposed to active or sham treatment and responses measured. Obviously, this design does not take into account all the environmental or the individual factors (genetic, lifestyle) that may influence drug availability, metabolism, and in general, pharmacological responses. According to the personalized medicine principles, every single individual is a universe. On the contrary, all the nuances and characteristics of individual patients are barely captured even by the most obsessive stratification of subjects from conventional trials. Alternative approaches do exist to tackle this issue and have been actively used in other areas of life sciences, yet seldom in medicine. Among those, it is worth citing the “N-of-1-trial” approach where the single individual is the object of the trial and responses are evaluated on an individual basis, rather than on a population/cohort basis [139, 140]. In N-of-1 trials, outcomes for treatments are compared within patients so that the optimal treatment for each subject can be established; comparisons are not carried between (or among) groups of patients, but rather each patient acts as his/her own control. However, N-of-1-trials may not be the panacea to solve any problem related to clinical trials. N-of-1-trials have cost issues and also the outcome should be clearly measurable, possibly in a short-time interval, as patients are repeatedly exposed to cycles of active or sham treatment and measures are taken at the end of each cycle. This clearly brings us back to the problem posed beforehand: what is the optimal response in SSc? Are short-term responses adequate to evaluate the long-term outcome a chronic disease? This paradigm has already emerged in SSc from conventional randomized controlled trials, where, for instance, cyclophosphamide has been associated with functional 12-month effects in subjects with interstitial lung disease but not long-term efficacy [141].

To gauge all the potential application and potentiality of precision medicine, including N-of-1-trials, we should however consider how these approaches may incorporate molecular data into patient care. A molecular stratification of patients may allow a better patient allocation to therapies, allowing the choice of the most appropriate treatment in relation to deregulated pathways and to biological mechanisms that contribute to disease phenotype. Previous work has already demonstrated that a population-based molecular stratification of SSc patients is feasible [125, 127], but this clearly does not meet the requisites of personalized medicine. Moreover, this approach may not be readily applicable to large-scale information from molecular medicine, as statistical power is obtained comparing one group against the other and this requires a large number of individuals. Thus, a number of potential pathways of interest may be overlooked because of inadequate sample size. A recently described framework, called “N-of-1-*pathways*,” has been proposed to overcome this issue and to focus on individual responses. Here, deregulated pathways from single individuals are analyzed and statistical power is obtained for a single patient with as few as two samples. This approach is feasible whenever two paired samples (healthy/diseased) are available from a single patient and as such is not applicable in all the disease. However, in SSc, this could be envisioned, as for instance when molecular fingertips from affected/unaffected skin samples are considered.

SSc remains a challenging disease to treat and the array of available molecules to treat scleroderma has not expanded much during the last decades. With the exception of therapies for the treatment of pulmonary hypertension, no recently discovered drug proved effective in SSc and is unequivocally recommended in the management of scleroderma [132]. The increasing knowledge about SSc pathogenesis and better patient allocation has however allowed a more suitable and effective use of existing therapies. New targeted drugs are currently being tested in phase 2 studies and are likely to change the future perspective of SSc treatment. Despite these encouraging premises, a lot of unanswered questions in the management of SSc still remain, and better and tailored therapies are likely to be discovered with the application of personalized medicine paradigms. This kind of approach is eagerly awaited by patients that need individualized therapies to solve their *own* problems besides numbers and statistics that are seldom of importance when it is *you* to be affected by a potentially devastating illness.

## Conclusion

### The Patient, Scientist and Clinician Strongly United: a Glance into the Future -the Search for a Cure

As discussed extensively in this review, SSc is a highly complex disease seen through the eyes of the patient, the scientist, as well as the clinician, justifying an integrated approach for the search of a cure. In line with what has becoming clearer in the eyes of the researcher and clinician, the journey of a patient with SSc is one that starts far before fibrosis becomes visible. As a matter of fact, it is likely to be characterized by a multitude of stressors that differs between patients in a temporal and spatial manner. In this light, it is tempting to speculate that this latter underlies the enormous heterogeneity in the clinical spectrum rather than the variation in genetic factors. This is underscored by the now widely accepted relative small contribution of genetic factors to chronic diseases including SSc which sheds light on the hypothesis that there is more than genetics alone. For instance, epigenetic factors are likely to play an essential role in the onset and perpetuation of SSc and other diseases. An individual’s epigenetic makeup is determined by multiple factors that one encounters during life. There is increasing interest in such factors which are all studied under the recently suggested concept of the exposome. To emphasize the importance of a more complete evaluation of environmental exposure, this concept called the exposome, which includes the entirety of environmental exposure from conception onwards, was introduced in 2005 [142]. The exposome consists of three overlapping domains: (1) the general external factors (socioeconomic), (2) specific external factors (lifestyle, occupations and pollutant exposure) and (3) internal factors (biological effects and response) [143]. As the exposome encapsulates time from conception to death, it needs to be measured multiple times in life by applying untargeted data-driven approaches in conjunction with computational modeling and computer learning techniques, which enables the reduction of dimension to make its outcomes useful for science and clinics.

**Fig. 1:**
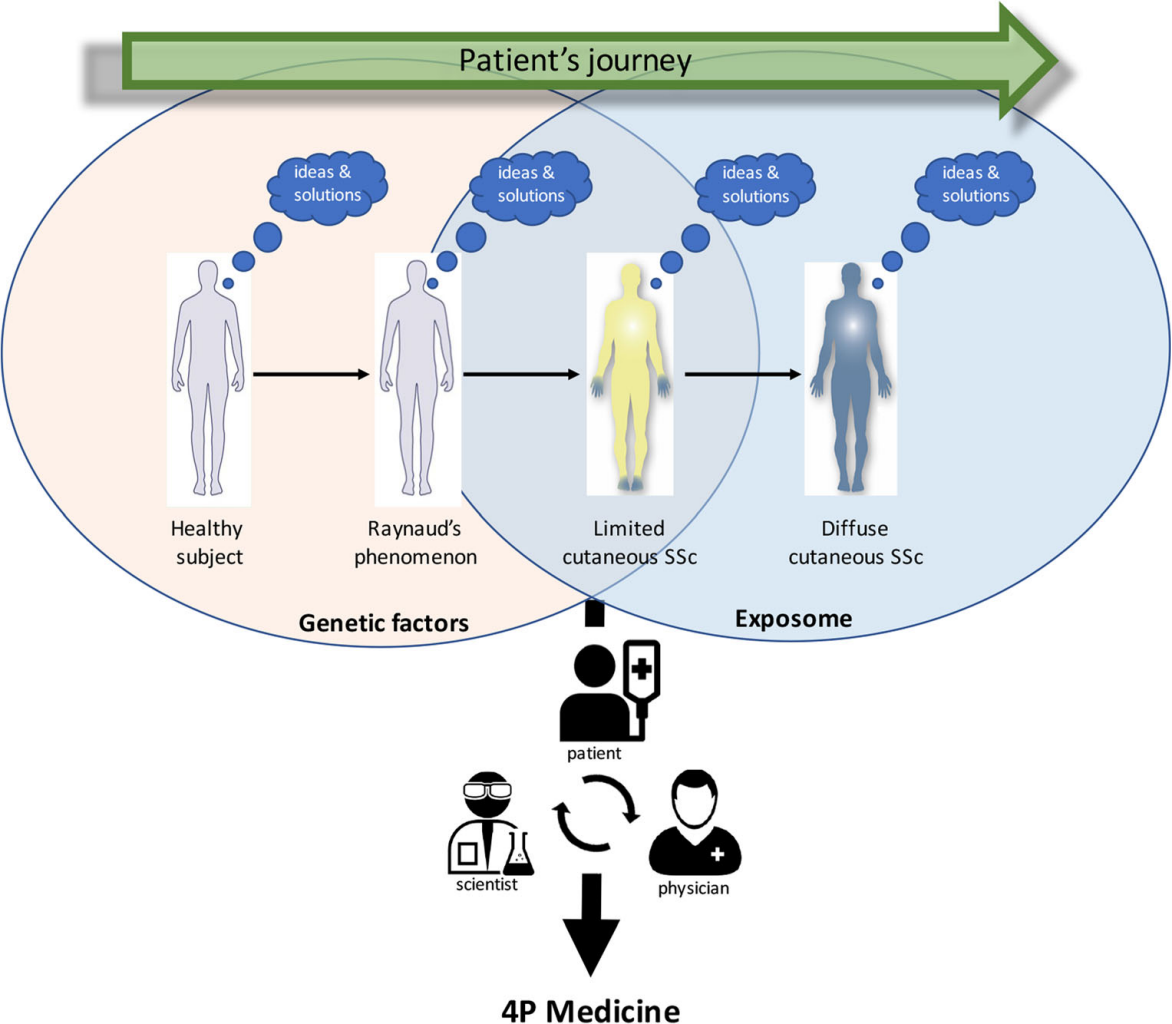
From clinical phenomena to predictive, preventative, personalized, and participatory (*4P*) medicine. During the patient’s journey, patients often have good ideas about the potential causes and complaints, how they have changed over time, why they have evolved, and how to deal with them. The interaction of patient-physician-scientists seems truly imperative in gaining molecular as well as clinical insights into the different phases (faces) of SSc. The future will tell whether a more integrated approach, taking into account ideas and suggestions from all three sources, will pave the way to 4P medicine

There is cumulating evidence for the role of the exposome in SSc considering the scientific reports on the toxic oil syndrome [144], the gadolinium [16], and silica exposure [12, 13] among others, and taking into account the anamneses taken from patients in daily practice. As discussed before, integrated analysis of multiple omics layers (genome, transcriptome, methylome, metabolome, microbiome, etc) is needed to understand the complex interactions between the exposome and clinical features. Ideally, such approaches should entail SSc patients having different phases of disease and encompass multiple cell subsets (stroma cells as well as immune cells) and embark on state-of-the-art computational analysis and computer learning models to truly understand the molecular changes leading up to the different aspects of SSc. Such approaches take a tremendous effort, but there are at least two studies undergoing -at least known to the authors -that encapsulate these thoughts. One is the Precisesads consortium (www.precisesads.eu) which investigates multiple omics layers from different clinical conditions including SSc by collecting biological samples from different centers throughout Europe. The other initiative -performed at the University Medical Center Utrecht (UMCU)— -focuses on various immune cell subsets— -including plasmacytoid dendritic cells, myeloid DC, monocytes, T cells, and B cells -from the circulation and tissues obtained from > 700 patients covering 12 immune-mediated inflammatory diseases. This cohort comprises now > 100 SSc patients in the different phases of diseases and is expected to bring forward the first results in the beginning of 2018. In an attempt to include all possible patient parameters— including the exposome, discussions with patients questioned in daily clinical practice as well as those participating in the sounding board of the UMCU are well integrated in data gathering. The aim is to capture and integrate exposome data in a holistic manner to understand SSc disease onset and progression in its full extent. Ultimately, we want to provide personalized care and disease intervention, for which the interaction of patient-physician-scientists seems truly imperative in gaining molecular as well as clinical insights into the different phases of SSc. The future will tell whether a more integrated approach, taking into account ideas and suggestions from all three sources, will pave the way to predictive, preventative, personalized, and participatory (4P) medicine (Fig. 1).

In conclusion, we hope to have highlighted all aspects of the disease from a patient’s, science’s and clinician’s perspective. It is clear that the ideas on how SSc arises, persists and progresses are very much alike. However, integration of these through the various molecular and epidemiological data sets into a patient-specific and, above all, targetable molecular network is the ultimate goal.

For that, patients, researchers and clinicians need to work even more closely together, united we stand.

#### A Patient’s View

I would like to make two final remarks:

- How nice it would be that there was one doctor who overviews all the different ways of SSc from the minute it presents itself or earlier.
- It gives me great confidence that there are such bright and intelligent people, who are capable of unraveling all this. The research to the answers we all need is in good hands.

## References

[1] Nikpour M, Baron M (2014). Mortality in systemic sclerosis: lessons learned from population-based and observational cohort studies. Curr Opin Rheumatol.

[2] Tyndall AJ, Bannert B, Vonk M, Airo P, Cozzi F, Carreira PE, Bancel DF, Allanore Y, Muller-Ladner U, Distler O, Iannone F, Pellerito R, Pileckyte M, Miniati I, Ananieva L, Gurman AB, Damjanov N, Mueller A, Valentini G, Riemekasten G, Tikly M, Hummers L, Henriques MJ, Caramaschi P, Scheja A, Rozman B, Ton E, Kumanovics G, Coleiro B, Feierl E, Szucs G, Von Muhlen CA, Riccieri V, Novak S, Chizzolini C, Kotulska A, Denton C, Coelho PC, Kotter I, Simsek I, de la Pena Lefebvre PG, Hachulla E, Seibold JR, Rednic S, Stork J, Morovic-Vergles J, Walker UA (2010). Causes and risk factors for death in systemic sclerosis: a study from the EULAR Scleroderma Trials and Research (EUSTAR) database. Ann Rheum Dis.

[3] Mouthon L, Alami S, Boisard AS, Chaigne B, Hachulla E, Poiraudeau S (2017). Patients’ views and needs about systemic sclerosis and its management: a qualitative interview study. BMC Musculoskelet Disord.

[4] Thombs BD, van Lankveld W, Bassel M, Baron M, Buzza R, Haslam S, Haythornthwaite JA, Hudson M, Jewett LR, Knafo R, Kwakkenbos L, Malcarne VL, Milette K, Motivala SJ, Newton EG, Nielson WR, Pacy M, Razykov I, Schieir O, Taillefer S, Worron-Sauve M (2010). Psychological health and well-being in systemic sclerosis: state of the science and consensus research agenda. Arthritis Care Res.

[5] Sumpton Daniel, Thakkar Vivek, O'Neill Sean, Singh-Grewal Davinder, Craig Jonathan C., Tong Allison (2017). “It's Not Me, It's Not Really Me.” Insights From Patients on Living With Systemic Sclerosis: An Interview Study. Arthritis Care & Research.

[6] Nakayama A, Tunnicliffe DJ, Thakkar V, Singh-Grewal D, O’Neill S, Craig JC, Tong A (2016). Patients’ perspectives and experiences living with systemic sclerosis: a systematic review and thematic synthesis of qualitative studies. J Rheumatol.

[7] van Tuyl LH, Boers M (2015). Patient-reported outcomes in core domain sets for rheumatic diseases. Nat Rev Rheumatol.

[8] Castrejon I, Gossec L, Carmona L (2015). The EULAR Outcome Measures Library: an evolutional database of validated patient-reported instruments. Ann Rheum Dis.

[9] Ingegnoli F, Carmona L, Castrejon I (2017). Systematic review of systemic sclerosis-specific instruments for the EULAR Outcome Measures Library: an evolutional database model of validated patient-reported outcomes. Semin Arthritis Rheum.

[10] Ho YY, Lagares D, Tager AM, Kapoor M (2014). Fibrosis—a lethal component of systemic sclerosis. Nat Rev Rheumatol.

[11] Broen JC, Radstake TR, Rossato M (2014). The role of genetics and epigenetics in the pathogenesis of systemic sclerosis. Nat Rev Rheumatol.

[12] McCormic ZD, Khuder SS, Aryal BK, Ames AL, Khuder SA (2010). Occupational silica exposure as a risk factor for scleroderma: a meta-analysis. Int Arch Occup Environ Health.

[13] Marie I, Gehanno JF, Bubenheim M, Duval-Modeste AB, Joly P, Dominique S, Bravard P, Noel D, Cailleux AF, Weber J, Lagoutte P, Benichou J, Levesque H (2014). Prospective study to evaluate the association between systemic sclerosis and occupational exposure and review of the literature. Autoimmun Rev.

[14] Kettaneh A, Al Moufti O, Tiev KP, Chayet C, Toledano C, Fabre B, Fardet L, Cabane J (2007). Occupational exposure to solvents and gender-related risk of systemic sclerosis: a metaanalysis of case-control studies. J Rheumatol.

[15] Nicholson WJ, Henneberger PK, Seidman H (1984). Occupational hazards in the VC-PVC industry. Prog Clin Biol Res.

[16] Cowper SE, Su LD, Bhawan J, Robin HS, LeBoit PE (2001). Nephrogenic fibrosing dermopathy. Am J Dermatopathol.

[17] Inaoki M, Kawabata C, Nishijima C, Yoshio N, Kita T (2012). Case of bleomycin-induced scleroderma. J Dermatol.

[18] Palestine RF, Millns JL, Spigel GT, Schroeter AL (1980). Skin manifestations of pentazocine abuse. J Am Acad Dermatol.

[19] Radic M, Kaliterna DM, Bonacin D, Vergles JM, Radic J, Fabijanic D, Kovacic V (2013). Is Helicobacter pylori infection a risk factor for disease severity in systemic sclerosis?. Rheumatol Int.

[20] Zakrzewska K, Corcioli F, Carlsen KM, Giuggioli D, Fanci R, Rinieri A, Ferri C, Azzi A (2009). Human parvovirus B19 (B19V) infection in systemic sclerosis patients. Intervirology.

[21] Magro CM, Nuovo G, Ferri C, Crowson AN, Giuggioli D, Sebastiani M (2004). Parvoviral infection of endothelial cells and stromal fibroblasts: a possible pathogenetic role in scleroderma. J Cutan Pathol.

[22] Lunardi C, Bason C, Navone R, Millo E, Damonte G, Corrocher R, Puccetti A (2000). Systemic sclerosis immunoglobulin G autoantibodies bind the human cytomegalovirus late protein UL94 and induce apoptosis in human endothelial cells. Nat Med.

[23] Farina A, Cirone M, York M, Lenna S, Padilla C, McLaughlin S, Faggioni A, Lafyatis R, Trojanowska M, Farina GA (2014). Epstein-Barr virus infection induces aberrant TLR activation pathway and fibroblast-myofibroblast conversion in scleroderma. J Invest Dermatol.

[24] Farina A, Peruzzi G, Lacconi V, Lenna S, Quarta S, Rosato E, Vestri AR, York M, Dreyfus DH, Faggioni A, Morrone S, Trojanowska M, Farina GA (2017). Epstein-Barr virus lytic infection promotes activation of Toll-like receptor 8 innate immune response in systemic sclerosis monocytes. Arthritis Res Ther.

[25] Abdollahi-Roodsaz S, Abramson SB, Scher JU (2016). The metabolic role of the gut microbiota in health and rheumatic disease: mechanisms and interventions. Nat Rev Rheumatol.

[26] Volkmann ER, Chang YL, Barroso N, Furst DE, Clements PJ, Gorn AH, Roth BE, Conklin JL, Getzug T, Borneman J, McGovern DP, Tong M, Jacobs JP, Braun J (2016). Association of systemic sclerosis with a unique colonic microbial consortium. Arthritis Rheumatol.

[27] Andreasson K, Alrawi Z, Persson A, Jonsson G, Marsal J (2016). Intestinal dysbiosis is common in systemic sclerosis and associated with gastrointestinal and extraintestinal features of disease. Arthritis Res Ther.

[28] Arora-Singh RK, Assassi S, del Junco DJ, Arnett FC, Perry M, Irfan U, Sharif R, Mattar T, Mayes MD (2010). Autoimmune diseases and autoantibodies in the first degree relatives of patients with systemic sclerosis. J Autoimmun.

[29] Feghali-Bostwick C, Medsger TA, Wright TM (2003). Analysis of systemic sclerosis in twins reveals low concordance for disease and high concordance for the presence of antinuclear antibodies. Arthritis Rheum.

[30] Bossini-Castillo L, Lopez-Isac E, Martin J (2015). Immunogenetics of systemic sclerosis: defining heritability, functional variants and shared-autoimmunity pathways. J Autoimmun.

[31] Radstake TR, Gorlova O, Rueda B, Martin JE, Alizadeh BZ, Palomino-Morales R, Coenen MJ, Vonk MC, Voskuyl AE, Schuerwegh AJ, Broen JC, van Riel PL, van’t Slot R, Italiaander A, Ophoff RA, Riemekasten G, Hunzelmann N, Simeon CP, Ortego-Centeno N, Gonzalez-Gay MA, Gonzalez-Escribano MF, Airo P, van Laar J, Herrick A, Worthington J, Hesselstrand R, Smith V, de Keyser F, Houssiau F, Chee MM, Madhok R, Shiels P, Westhovens R, Kreuter A, Kiener H, de Baere E, Witte T, Padykov L, Klareskog L, Beretta L, Scorza R, Lie BA, Hoffmann-Vold AM, Carreira P, Varga J, Hinchcliff M, Gregersen PK, Lee AT, Ying J, Han Y, Weng SF, Amos CI, Wigley FM, Hummers L, Nelson JL, Agarwal SK, Assassi S, Gourh P, Tan FK, Koeleman BP, Arnett FC, Martin J, Mayes MD, Spanish Scleroderma G (2010). Genome-wide association study of systemic sclerosis identifies CD247 as a new susceptibility locus. Nat Genet.

[32] Mayes MD, Bossini-Castillo L, Gorlova O, Martin JE, Zhou X, Chen WV, Assassi S, Ying J, Tan FK, Arnett FC, Reveille JD, Guerra S, Teruel M, Carmona FD, Gregersen PK, Lee AT, Lopez-Isac E, Ochoa E, Carreira P, Simeon CP, Castellvi I, Gonzalez-Gay MA, Spanish Scleroderma G, Zhernakova A, Padyukov L, Alarcon-Riquelme M, Wijmenga C, Brown M, Beretta L, Riemekasten G, Witte T, Hunzelmann N, Kreuter A, Distler JH, Voskuyl AE, Schuerwegh AJ, Hesselstrand R, Nordin A, Airo P, Lunardi C, Shiels P, van Laar JM, Herrick A, Worthington J, Denton C, Wigley FM, Hummers LK, Varga J, Hinchcliff ME, Baron M, Hudson M, Pope JE, Furst DE, Khanna D, Phillips K, Schiopu E, Segal BM, Molitor JA, Silver RM, Steen VD, Simms RW, Lafyatis RA, Fessler BJ, Frech TM, Alkassab F, Docherty P, Kaminska E, Khalidi N, Jones HN, Markland J, Robinson D, Broen J, Radstake TR, Fonseca C, Koeleman BP, Martin J (2014). Immunochip analysis identifies multiple susceptibility loci for systemic sclerosis. Am J Hum Genet.

[33] Ruzehaji N, Frantz C, Ponsoye M, Avouac J, Pezet S, Guilbert T, Luccarini JM, Broqua P, Junien JL, Allanore Y (2016). Pan PPAR agonist IVA337 is effective in prevention and treatment of experimental skin fibrosis. Ann Rheum Dis.

[34] Wang YY, Wang Q, Sun XH, Liu RZ, Shu Y, Kanekura T, Huang JH, Li YP, Wang JC, Zhao M, Lu QJ, Xiao R (2014). DNA hypermethylation of the forkhead box protein 3 (FOXP3) promoter in CD4+ T cells of patients with systemic sclerosis. Br J Dermatol.

[35] Gilbane AJ, Derrett-Smith E, Trinder SL, Good RB, Pearce A, Denton CP, Holmes AM (2015). Impaired bone morphogenetic protein receptor II signaling in a transforming growth factor-beta-dependent mouse model of pulmonary hypertension and in systemic sclerosis. Am J Respir Crit Care Med.

[36] Wang Y, Kahaleh B (2013). Epigenetic repression of bone morphogenetic protein receptor II expression in scleroderma. J Cell Mol Med.

[37] Rossato Marzia, Affandi Alsya J., Thordardottir Soley, Wichers Catharina G. K., Cossu Marta, Broen Jasper C. A., Moret Frederique M., Bossini-Castillo Lara, Chouri Eleni, van Bon Lenny, Wolters Femke, Marut Wioleta, van der Kroef Maarten, Silva-Cardoso Sandra, Bekker Cornelis P. J., Dolstra Harry, van Laar Jacob M., Martin Javier, van Roon Joel A. G., Reedquist Kris A., Beretta Lorenzo, Radstake Timothy R. D. J. (2017). Association of MicroRNA-618 Expression With Altered Frequency and Activation of Plasmacytoid Dendritic Cells in Patients With Systemic Sclerosis. Arthritis & Rheumatology.

[38] Brkic Z, van Bon L, Cossu M, van Helden-Meeuwsen CG, Vonk MC, Knaapen H, van den Berg W, Dalm VA, Van Daele PL, Severino A, Maria NI, Guillen S, Dik WA, Beretta L, Versnel MA, Radstake T (2016). The interferon type I signature is present in systemic sclerosis before overt fibrosis and might contribute to its pathogenesis through high BAFF gene expression and high collagen synthesis. Ann Rheum Dis.

[39] Campbell PM, LeRoy EC (1975). Pathogenesis of systemic sclerosis: a vascular hypothesis. Semin Arthritis Rheum.

[40] Prescott RJ, Freemont AJ, Jones CJ, Hoyland J, Fielding P (1992). Sequential dermal microvascular and perivascular changes in the development of scleroderma. J Pathol.

[41] Andersen GN, Caidahl K, Kazzam E, Petersson AS, Waldenstrom A, Mincheva-Nilsson L, Rantapaa-Dahlqvist S (2000). Correlation between increased nitric oxide production and markers of endothelial activation in systemic sclerosis: findings with the soluble adhesion molecules E-selectin, intercellular adhesion molecule 1, and vascular cell adhesion molecule 1. Arthritis Rheum.

[42] Gruschwitz MS, Hornstein OP, von Den Driesch P (1995). Correlation of soluble adhesion molecules in the peripheral blood of scleroderma patients with their in situ expression and with disease activity. Arthritis Rheum.

[43] Rabquer BJ, Hou Y, Del Galdo F, Kenneth Haines G, Gerber ML, Jimenez SA, Seibold JR, Koch AE (2009). The proadhesive phenotype of systemic sclerosis skin promotes myeloid cell adhesion via ICAM-1 and VCAM-1. Rheumatology (Oxford).

[44] Vancheeswaran R, Magoulas T, Efrat G, Wheeler-Jones C, Olsen I, Penny R, Black CM (1994). Circulating endothelin-1 levels in systemic sclerosis subsets—a marker of fibrosis or vascular dysfunction?. J Rheumatol.

[45] Cotton SA, Herrick AL, Jayson MI, Freemont AJ (1999). Endothelial expression of nitric oxide synthases and nitrotyrosine in systemic sclerosis skin. J Pathol.

[46] Sambo P, Baroni SS, Luchetti M, Paroncini P, Dusi S, Orlandini G, Gabrielli A (2001). Oxidative stress in scleroderma: maintenance of scleroderma fibroblast phenotype by the constitutive up-regulation of reactive oxygen species generation through the NADPH oxidase complex pathway. Arthritis Rheum.

[47] Distler O, Del Rosso A, Giacomelli R, Cipriani P, Conforti ML, Guiducci S, Gay RE, Michel BA, Bruhlmann P, Muller-Ladner U, Gay S, Matucci-Cerinic M (2002). Angiogenic and angiostatic factors in systemic sclerosis: increased levels of vascular endothelial growth factor are a feature of the earliest disease stages and are associated with the absence of fingertip ulcers. Arthritis Res.

[48] Distler O, Distler JH, Scheid A, Acker T, Hirth A, Rethage J, Michel BA, Gay RE, Muller-Ladner U, Matucci-Cerinic M, Plate KH, Gassmann M, Gay S (2004). Uncontrolled expression of vascular endothelial growth factor and its receptors leads to insufficient skin angiogenesis in patients with systemic sclerosis. Circ Res.

[49] Avouac J, Wipff J, Goldman O, Ruiz B, Couraud PO, Chiocchia G, Kahan A, Boileau C, Uzan G, Allanore Y (2008). Angiogenesis in systemic sclerosis: impaired expression of vascular endothelial growth factor receptor 1 in endothelial progenitor-derived cells under hypoxic conditions. Arthritis Rheum.

[50] Manetti M, Guiducci S, Romano E, Ceccarelli C, Bellando-Randone S, Conforti ML, Ibba-Manneschi L, Matucci-Cerinic M (2011). Overexpression of VEGF165b, an inhibitory splice variant of vascular endothelial growth factor, leads to insufficient angiogenesis in patients with systemic sclerosis. Circ Res.

[51] Del Papa N, Quirici N, Soligo D, Scavullo C, Cortiana M, Borsotti C, Maglione W, Comina DP, Vitali C, Fraticelli P, Gabrielli A, Cortelezzi A, Lambertenghi-Deliliers G (2006). Bone marrow endothelial progenitors are defective in systemic sclerosis. Arthritis Rheum.

[52] LeRoy EC, Medsger TA (2001). Criteria for the classification of early systemic sclerosis. J Rheumatol.

[53] Valentini G, Marcoccia A, Cuomo G, Vettori S, Iudici M, Bondanini F, Santoriello C, Ciani A, Cozzolino D, De Matteis GM, Cappabianca S, Vitelli F, Spano A (2013). Early systemic sclerosis: marker autoantibodies and videocapillaroscopy patterns are each associated with distinct clinical, functional and cellular activation markers. Arthritis Res Ther.

[54] Koenig M, Joyal F, Fritzler MJ, Roussin A, Abrahamowicz M, Boire G, Goulet JR, Rich E, Grodzicky T, Raymond Y, Senecal JL (2008). Autoantibodies and microvascular damage are independent predictive factors for the progression of Raynaud’s phenomenon to systemic sclerosis: a twenty-year prospective study of 586 patients, with validation of proposed criteria for early systemic sclerosis. Arthritis Rheum.

[55] Vigone B, Santaniello A, Marchini M, Montanelli G, Caronni M, Severino A, Beretta L (2015). Role of class II human leucocyte antigens in the progression from early to definite systemic sclerosis. Rheumatology (Oxford).

[56] van den Hoogen F, Khanna D, Fransen J, Johnson SR, Baron M, Tyndall A, Matucci-Cerinic M, Naden RP, Medsger TA, Carreira PE, Riemekasten G, Clements PJ, Denton CP, Distler O, Allanore Y, Furst DE, Gabrielli A, Mayes MD, van Laar JM, Seibold JR, Czirjak L, Steen VD, Inanc M, Kowal-Bielecka O, Muller-Ladner U, Valentini G, Veale DJ, Vonk MC, Walker UA, Chung L, Collier DH, Ellen Csuka M, Fessler BJ, Guiducci S, Herrick A, Hsu VM, Jimenez S, Kahaleh B, Merkel PA, Sierakowski S, Silver RM, Simms RW, Varga J, Pope JE (2013). 2013 Classification criteria for systemic sclerosis: an American College of Rheumatology/European League Against Rheumatism Collaborative Initiative. Ann Rheum Dis.

[57] Vettori S, Cuomo G, Iudici M, D’Abrosca V, Giacco V, Barra G, De Palma R, Valentini G (2014). Early systemic sclerosis: serum profiling of factors involved in endothelial, T-cell, and fibroblast interplay is marked by elevated interleukin-33 levels. J Clin Immunol.

[58] Cossu M, Andracco R, Santaniello A, Marchini M, Severino A, Caronni M, Radstake T, Beretta L (2016). Serum levels of vascular dysfunction markers reflect disease severity and stage in systemic sclerosis patients. Rheumatology (Oxford).

[59] Sgonc R, Gruschwitz MS, Dietrich H, Recheis H, Gershwin ME, Wick G (1996). Endothelial cell apoptosis is a primary pathogenetic event underlying skin lesions in avian and human scleroderma. J Clin Invest.

[60] Sakkas LI, Xu B, Artlett CM, Lu S, Jimenez SA, Platsoucas CD (2002). Oligoclonal T cell expansion in the skin of patients with systemic sclerosis. J Immunol.

[61] Roumm AD, Whiteside TL, Medsger TA, Rodnan GP (1984). Lymphocytes in the skin of patients with progressive systemic sclerosis. Quantification, subtyping, and clinical correlations. Arthritis Rheum.

[62] Fuschiotti P, Larregina AT, Ho J, Feghali-Bostwick C, Medsger TA (2013). Interleukin-13-producing CD8+ T cells mediate dermal fibrosis in patients with systemic sclerosis. Arthritis Rheum.

[63] Kraling BM, Maul GG, Jimenez SA (1995). Mononuclear cellular infiltrates in clinically involved skin from patients with systemic sclerosis of recent onset predominantly consist of monocytes/macrophages. Pathobiology.

[64] Kalogerou A, Gelou E, Mountantonakis S, Settas L, Zafiriou E, Sakkas L (2005). Early T cell activation in the skin from patients with systemic sclerosis. Ann Rheum Dis.

[65] Giacomelli R, Matucci-Cerinic M, Cipriani P, Ghersetich I, Lattanzio R, Pavan A, Pignone A, Cagnoni ML, Lotti T, Tonietti G (1998). Circulating Vdelta1+ T cells are activated and accumulate in the skin of systemic sclerosis patients. Arthritis Rheum.

[66] Klein S, Kretz CC, Ruland V, Stumpf C, Haust M, Hartschuh W, Hartmann M, Enk A, Suri-Payer E, Oberle N, Krammer PH, Kuhn A (2011). Reduction of regulatory T cells in skin lesions but not in peripheral blood of patients with systemic scleroderma. Ann Rheum Dis.

[67] Sgonc R, Gruschwitz MS, Boeck G, Sepp N, Gruber J, Wick G (2000). Endothelial cell apoptosis in systemic sclerosis is induced by antibody-dependent cell-mediated cytotoxicity via CD95. Arthritis Rheum.

[68] Waldman WJ, Knight DA, Adams PW (1998). Cytolytic activity against allogeneic human endothelia: resistance of cytomegalovirus-infected cells and virally activated lysis of uninfected cells. Transplantation.

[69] York MR, Nagai T, Mangini AJ, Lemaire R, van Seventer JM, Lafyatis R (2007). A macrophage marker, Siglec-1, is increased on circulating monocytes in patients with systemic sclerosis and induced by type I interferons and toll-like receptor agonists. Arthritis Rheum.

[70] Higgs BW, Liu Z, White B, Zhu W, White WI, Morehouse C, Brohawn P, Kiener PA, Richman L, Fiorentino D, Greenberg SA, Jallal B, Yao Y (2011). Patients with systemic lupus erythematosus, myositis, rheumatoid arthritis and scleroderma share activation of a common type I interferon pathway. Ann Rheum Dis.

[71] van Bon L, Affandi AJ, Broen J, Christmann RB, Marijnissen RJ, Stawski L, Farina GA, Stifano G, Mathes AL, Cossu M, York M, Collins C, Wenink M, Huijbens R, Hesselstrand R, Saxne T, DiMarzio M, Wuttge D, Agarwal SK, Reveille JD, Assassi S, Mayes M, Deng Y, Drenth JP, de Graaf J, den Heijer M, Kallenberg CG, Bijl M, Loof A, van den Berg WB, Joosten LA, Smith V, de Keyser F, Scorza R, Lunardi C, van Riel PL, Vonk M, van Heerde W, Meller S, Homey B, Beretta L, Roest M, Trojanowska M, Lafyatis R, Radstake TR (2014). Proteome-wide analysis and CXCL4 as a biomarker in systemic sclerosis. N Engl J Med.

[72] van Bon L, Cossu M, Radstake TR (2011). An update on an immune system that goes awry in systemic sclerosis. Curr Opin Rheumatol.

[73] van Bon L, Popa C, Huijbens R, Vonk M, York M, Simms R, Hesselstrand R, Wuttge DM, Lafyatis R, Radstake TR (2010). Distinct evolution of TLR-mediated dendritic cell cytokine secretion in patients with limited and diffuse cutaneous systemic sclerosis. Ann Rheum Dis.

[74] Roelofs MF, Joosten LA, Abdollahi-Roodsaz S, van Lieshout AW, Sprong T, van den Hoogen FH, van den Berg WB, Radstake TR (2005). The expression of toll-like receptors 3 and 7 in rheumatoid arthritis synovium is increased and costimulation of toll-like receptors 3, 4, and 7/8 results in synergistic cytokine production by dendritic cells. Arthritis Rheum.

[75] Marshak-Rothstein A (2006). Toll-like receptors in systemic autoimmune disease. Nat Rev Immunol.

[76] Kurasawa K, Hirose K, Sano H, Endo H, Shinkai H, Nawata Y, Takabayashi K, Iwamoto I (2000). Increased interleukin-17 production in patients with systemic sclerosis. Arthritis Rheum.

[77] Truchetet ME, Brembilla NC, Montanari E, Lonati P, Raschi E, Zeni S, Fontao L, Meroni PL, Chizzolini C (2013). Interleukin-17A+ cell counts are increased in systemic sclerosis skin and their number is inversely correlated with the extent of skin involvement. Arthritis Rheum.

[78] Brembilla NC, Montanari E, Truchetet ME, Raschi E, Meroni P, Chizzolini C (2013). Th17 cells favor inflammatory responses while inhibiting type I collagen deposition by dermal fibroblasts: differential effects in healthy and systemic sclerosis fibroblasts. Arthritis Res Ther.

[79] Mavalia C, Scaletti C, Romagnani P, Carossino AM, Pignone A, Emmi L, Pupilli C, Pizzolo G, Maggi E, Romagnani S (1997). Type 2 helper T-cell predominance and high CD30 expression in systemic sclerosis. Am J Pathol.

[80] Parel Y, Aurrand-Lions M, Scheja A, Dayer JM, Roosnek E, Chizzolini C (2007). Presence of CD4+CD8+ double-positive T cells with very high interleukin-4 production potential in lesional skin of patients with systemic sclerosis. Arthritis Rheum.

[81] Sandler NG, Mentink-Kane MM, Cheever AW, Wynn TA (2003). Global gene expression profiles during acute pathogen-induced pulmonary inflammation reveal divergent roles for Th1 and Th2 responses in tissue repair. J Immunol.

[82] Valentini G, Baroni A, Esposito K, Naclerio C, Buommino E, Farzati A, Cuomo G, Farzati B (2001). Peripheral blood T lymphocytes from systemic sclerosis patients show both Th1 and Th2 activation. J Clin Immunol.

[83] Radstake TR, van Bon L, Broen J, Wenink M, Santegoets K, Deng Y, Hussaini A, Simms R, Cruikshank WW, Lafyatis R (2009). Increased frequency and compromised function of T regulatory cells in systemic sclerosis (SSc) is related to a diminished CD69 and TGFbeta expression. PLoS One.

[84] Horikawa M, Hasegawa M, Komura K, Hayakawa I, Yanaba K, Matsushita T, Takehara K, Sato S (2005). Abnormal natural killer cell function in systemic sclerosis: altered cytokine production and defective killing activity. J Invest Dermatol.

[85] Cossu M, van Bon L, Nierkens S, Bellocchi C, Santaniello A, Dolstra H, Beretta L, Radstake TR (2016). The magnitude of cytokine production by stimulated CD56+ cells is associated with early stages of systemic sclerosis. Clin Immunol.

[86] Almeida I, Silva SV, Fonseca AR, Silva I, Vasconcelos C, Lima M (2015). T and NK cell phenotypic abnormalities in systemic sclerosis: a cohort study and a comprehensive literature review. Clin Rev Allergy Immunol.

[87] Worda M, Sgonc R, Dietrich H, Niederegger H, Sundick RS, Gershwin ME, Wick G (2003). In vivo analysis of the apoptosis-inducing effect of anti-endothelial cell antibodies in systemic sclerosis by the chorionallantoic membrane assay. Arthritis Rheum.

[88] Kim D, Peck A, Santer D, Patole P, Schwartz SM, Molitor JA, Arnett FC, Elkon KB (2008). Induction of interferon-alpha by scleroderma sera containing autoantibodies to topoisomerase I: association of higher interferon-alpha activity with lung fibrosis. Arthritis Rheum.

[89] Villani Alexandra-Chloé, Satija Rahul, Reynolds Gary, Sarkizova Siranush, Shekhar Karthik, Fletcher James, Griesbeck Morgane, Butler Andrew, Zheng Shiwei, Lazo Suzan, Jardine Laura, Dixon David, Stephenson Emily, Nilsson Emil, Grundberg Ida, McDonald David, Filby Andrew, Li Weibo, De Jager Philip L., Rozenblatt-Rosen Orit, Lane Andrew A., Haniffa Muzlifah, Regev Aviv, Hacohen Nir (2017). Single-cell RNA-seq reveals new types of human blood dendritic cells, monocytes, and progenitors. Science.

[90] Rieckmann JC, Geiger R, Hornburg D, Wolf T, Kveler K, Jarrossay D, Sallusto F, Shen-Orr SS, Lanzavecchia A, Mann M, Meissner F (2017). Social network architecture of human immune cells unveiled by quantitative proteomics. Nat Immunol.

[91] Notta F, Zandi S, Takayama N, Dobson S, Gan OI, Wilson G, Kaufmann KB, McLeod J, Laurenti E, Dunant CF, McPherson JD, Stein LD, Dror Y, Dick JE (2016). Distinct routes of lineage development reshape the human blood hierarchy across ontogeny. Science.

[92] Javierre BM, Burren OS, Wilder SP, Kreuzhuber R, Hill SM, Sewitz S, Cairns J, Wingett SW, Varnai C, Thiecke MJ, Burden F, Farrow S, Cutler AJ, Rehnstrom K, Downes K, Grassi L, Kostadima M, Freire-Pritchett P, Wang F, Consortium B, Stunnenberg HG, Todd JA, Zerbino DR, Stegle O, Ouwehand WH, Frontini M, Wallace C, Spivakov M, Fraser P (2016). Lineage-specific genome architecture links enhancers and non-coding disease variants to target gene promoters. Cell.

[93] Kallenberg CG (1991). Connective tissue disease in patients presenting with Raynaud’s phenomenon alone. Ann Rheum Dis.

[94] Maverakis E, Patel F, Kronenberg DG, Chung L, Fiorentino D, Allanore Y, Guiducci S, Hesselstrand R, Hummers LK, Duong C, Kahaleh B, Macgregor A, Matucci-Cerinic M, Wollheim FA, Mayes MD, Gershwin ME (2014). International consensus criteria for the diagnosis of Raynaud’s phenomenon. J Autoimmun.

[95] Herrick AL, Cutolo M (2010). Clinical implications from capillaroscopic analysis in patients with Raynaud's phenomenon and systemic sclerosis. Arthritis Rheum.

[96] Cutolo M, Sulli A, Pizzorni C, Accardo S (2000). Nailfold videocapillaroscopy assessment of microvascular damage in systemic sclerosis. J Rheumatol.

[97] Wigley FM (2001). When is scleroderma really scleroderma?. J Rheumatol.

[98] Ingegnoli F, Boracchi P, Gualtierotti R, Biganzoli EM, Zeni S, Lubatti C, Fantini F (2010). Improving outcome prediction of systemic sclerosis from isolated Raynaud’s phenomenon: role of autoantibodies and nail-fold capillaroscopy. Rheumatology (Oxford).

[99] Valentini G, Marcoccia A, Cuomo G, Vettori S, Iudici M, Bondanini F, Santoriello C, Ciani A, Cozzolino D, De Matteis GM, Cappabianca S, Vitelli F, Spano A (2014). Early systemic sclerosis: analysis of the disease course in patients with marker autoantibody and/or capillaroscopic positivity. Arthritis Care Res.

[100] Masi Alfonse T. (1980). Preliminary criteria for the classification of systemic sclerosis (scleroderma). Arthritis & Rheumatism.

[101] Ho KT, Reveille JD (2003). The clinical relevance of autoantibodies in scleroderma. Arthritis Res Ther.

[102] Lonzetti LS, Joyal F, Raynauld JP, Roussin A, Goulet JR, Rich E, Choquette D, Raymond Y, Senecal JL (2001). Updating the American College of Rheumatology preliminary classification criteria for systemic sclerosis: addition of severe nailfold capillaroscopy abnormalities markedly increases the sensitivity for limited scleroderma. Arthritis Rheum.

[103] Jordan S, Maurer B, Toniolo M, Michel B, Distler O (2015). Performance of the new ACR/EULAR classification criteria for systemic sclerosis in clinical practice. Rheumatology (Oxford).

[104] Alhajeri H, Hudson M, Fritzler M, Pope J, Tatibouet S, Markland J, Robinson D, Jones N, Khalidi N, Docherty P, Kaminska E, Masetto A, Sutton E, Mathieu JP, Ligier S, Grodzicky T, LeClercq S, Thorne C, Gyger G, Smith D, Fortin PR, Larche M, Baron M (2015). 2013 American College of Rheumatology/European League against rheumatism classification criteria for systemic sclerosis outperform the 1980 criteria: data from the Canadian Scleroderma Research Group. Arthritis Care Res.

[105] Steen VD, Medsger TA (2000). Severe organ involvement in systemic sclerosis with diffuse scleroderma. Arthritis Rheum.

[106] Beretta L, Santaniello A, Cappiello F, Chawla NV, Vonk MC, Carreira PE, Allanore Y, Popa-Diaconu DA, Cossu M, Bertolotti F, Ferraccioli G, Mazzone A, Scorza R (2010). Development of a five-year mortality model in systemic sclerosis patients by different analytical approaches. Clin Exp Rheumatol.

[107] Cutolo M, Sulli A, Smith V (2010). Assessing microvascular changes in systemic sclerosis diagnosis and management. Nat Rev Rheumatol.

[108] Sulli A, Pizzorni C, Smith V, Zampogna G, Ravera F, Cutolo M (2012). Timing of transition between capillaroscopic patterns in systemic sclerosis. Arthritis Rheum.

[109] Caramaschi P, Canestrini S, Martinelli N, Volpe A, Pieropan S, Ferrari M, Bambara LM, Carletto A, Biasi D (2007). Scleroderma patients nailfold videocapillaroscopic patterns are associated with disease subset and disease severity. Rheumatology (Oxford).

[110] Smith V, Riccieri V, Pizzorni C, Decuman S, Deschepper E, Bonroy C, Sulli A, Piette Y, De Keyser F, Cutolo M (2013). Nailfold capillaroscopy for prediction of novel future severe organ involvement in systemic sclerosis. J Rheumatol.

[111] Soulaidopoulos Stergios, Triantafyllidou Eva, Garyfallos Alexandros, Kitas George D., Dimitroulas Theodoros (2017). The role of nailfold capillaroscopy in the assessment of internal organ involvement in systemic sclerosis: A critical review. Autoimmunity Reviews.

[112] Smith V, Decuman S, Sulli A, Bonroy C, Piettte Y, Deschepper E, de Keyser F, Cutolo M (2012). Do worsening scleroderma capillaroscopic patterns predict future severe organ involvement? A pilot study. Ann Rheum Dis.

[113] Medsger TA, Bombardieri S, Czirjak L, Scorza R, Della Rossa A, Bencivelli W (2003). Assessment of disease severity and prognosis. Clin Exp Rheumatol.

[114] Smith V, De Keyser F, Pizzorni C, Van Praet JT, Decuman S, Sulli A, Deschepper E, Cutolo M (2011). Nailfold capillaroscopy for day-to-day clinical use: construction of a simple scoring modality as a clinical prognostic index for digital trophic lesions. Ann Rheum Dis.

[115] Cutolo M, Herrick AL, Distler O, Becker MO, Beltran E, Carpentier P, Ferri C, Inanc M, Vlachoyiannopoulos P, Chadha-Boreham H, Cottreel E, Pfister T, Rosenberg D, Torres JV, Smith V, Investigators CAPS (2016). Nailfold videocapillaroscopic features and other clinical risk factors for digital ulcers in systemic sclerosis: a multicenter, prospective cohort study. Arthritis Rheumatol.

[116] LeRoy EC, Black C, Fleischmajer R, Jablonska S, Krieg T, Medsger TA, Rowell N, Wollheim F (1988). Scleroderma (systemic sclerosis): classification, subsets and pathogenesis. J Rheumatol.

[117] Walker UA, Tyndall A, Czirjak L, Denton C, Farge-Bancel D, Kowal-Bielecka O, Muller-Ladner U, Bocelli-Tyndall C, Matucci-Cerinic M (2007). Clinical risk assessment of organ manifestations in systemic sclerosis: a report from the EULAR Scleroderma Trials and Research group database. Ann Rheum Dis.

[118] Muangchan C, Harding S, Khimdas S, Bonner A, Baron M, Pope J, Canadian Scleroderma Research G (2012). Association of C-reactive protein with high disease activity in systemic sclerosis: results from the Canadian Scleroderma Research Group. Arthritis Care Res.

[119] Medsger TA (2003). Natural history of systemic sclerosis and the assessment of disease activity, severity, functional status, and psychologic well-being. Rheum Dis Clin N Am.

[120] van den Hombergh WM, Carreira PE, Knaapen-Hans HK, van den Hoogen FH, Fransen J, Vonk MC (2016). An easy prediction rule for diffuse cutaneous systemic sclerosis using only the timing and type of first symptoms and auto-antibodies: derivation and validation. Rheumatology (Oxford).

[121] Domsic RT, Nihtyanova SI, Wisniewski SR, Fine MJ, Lucas M, Kwoh CK, Denton CP, Medsger TA (2016). Derivation and external validation of a prediction rule for five-year mortality in patients with early diffuse cutaneous systemic sclerosis. Arthritis Rheumatol.

[122] Domsic RT, Nihtyanova SI, Wisniewski SR, Fine MJ, Lucas M, Kwoh CK, Denton CP, Medsger TA (2014). Derivation and validation of a prediction rule for two-year mortality in early diffuse cutaneous systemic sclerosis. Arthritis Rheumatol.

[123] Teruel M, Chamberlain C, Alarcon-Riquelme ME (2017). Omics studies: their use in diagnosis and reclassification of SLE and other systemic autoimmune diseases. Rheumatology (Oxford).

[124] Assassi S, Wu M, Tan FK, Chang J, Graham TA, Furst DE, Khanna D, Charles J, Ferguson EC, Feghali-Bostwick C, Mayes MD (2013). Skin gene expression correlates of severity of interstitial lung disease in systemic sclerosis. Arthritis Rheum.

[125] Lofgren S, Hinchcliff M, Carns M, Wood T, Aren K, Arroyo E, Cheung P, Kuo A, Valenzuela A, Haemel A, Wolters PJ, Gordon J, Spiera R, Assassi S, Boin F, Chung L, Fiorentino D, Utz PJ, Whitfield ML, Khatri P (2016). Integrated, multicohort analysis of systemic sclerosis identifies robust transcriptional signature of disease severity. JCI Insight.

[126] Haddon DJ, Wand HE, Jarrell JA, Spiera RF, Utz PJ, Gordon JK, Chung LS (2017). Proteomic analysis of sera from individuals with diffuse cutaneous systemic sclerosis reveals a multianalyte signature associated with clinical improvement during imatinib mesylate treatment. J Rheumatol.

[127] Volkmann ER, Tashkin DP, Roth MD, Clements PJ, Khanna D, Furst DE, Mayes M, Charles J, Tseng CH, Elashoff RM, Assassi S (2016). Changes in plasma CXCL4 levels are associated with improvements in lung function in patients receiving immunosuppressive therapy for systemic sclerosis-related interstitial lung disease. Arthritis Res Ther.

[128] Valentini G, Iudici M, Walker UA, Jaeger VK, Baron M, Carreira P, Czirjak L, Denton CP, Distler O, Hachulla E, Herrick AL, Kowal-Bielecka O, Pope J, Muller-Ladner U, Riemekasten G, Avouac J, Frerix M, Jordan S, Minier T, Siegert E, Ong VH, Vettori S, Allanore Y (2017). The European Scleroderma Trials and Research group (EUSTAR) task force for the development of revised activity criteria for systemic sclerosis: derivation and validation of a preliminarily revised EUSTAR activity index. Ann Rheum Dis.

[129] Khanna D, Berrocal VJ, Giannini EH, Seibold JR, Merkel PA, Mayes MD, Baron M, Clements PJ, Steen V, Assassi S, Schiopu E, Phillips K, Simms RW, Allanore Y, Denton CP, Distler O, Johnson SR, Matucci-Cerinic M, Pope JE, Proudman SM, Siegel J, Wong WK, Wells AU, Furst DE (2016). The American College of Rheumatology provisional composite response index for clinical trials in early diffuse cutaneous systemic sclerosis. Arthritis Rheumatol.

[130] Riehm KE, Kwakkenbos L, Carrier ME, Bartlett SJ, Malcarne VL, Mouthon L, Nielson WR, Poiraudeau S, Nielsen K, Baron M, Frech T, Hudson M, Pope J, Sauve M, Suarez-Almazor ME, Wigley FM, Thombs BD, Scleroderma Patient-Centered Intervention Network I (2016). Validation of the self-efficacy for managing chronic disease scale: a scleroderma patient-centered intervention network cohort study. Arthritis Care Res.

[131] Avouac J, Kowal-Bielecka O, Landewe R, Chwiesko S, Miniati I, Czirjak L, Clements P, Denton C, Farge D, Fligelstone K, Földvari I, Furst DE, Müller-Ladner U, Seibold J, Silver RM, Takehara K, Toth BG, Tyndall A, Valentini G, van den Hoogen F, Wigley F, Zulian F, Matucci-Cerinic M, EUSTAR Coauthors (2009). European League Against Rheumatism (EULAR) Scleroderma Trial and Research group (EUSTAR) recommendations for the treatment of systemic sclerosis: methods of elaboration and results of systematic literature research. Ann Rheum Dis.

[132] Kowal-Bielecka O, Fransen J, Avouac J, Becker M, Kulak A, Allanore Y, Distler O, Clements P, Cutolo M, Czirjak L, Damjanov N, Del Galdo F, Denton CP, JHW D, Foeldvari I, Figelstone K, Frerix M, Furst DE, Guiducci S, Hunzelmann N, Khanna D, Matucci-Cerinic M, Herrick AL, van den Hoogen F, van Laar JM, Riemekasten G, Silver R, Smith V, Sulli A, Tarner I, Tyndall A, Welling J, Wigley F, Valentini G, Walker UA, Zulian F, Müller-Ladner U, EUSTAR Coauthors (2017). Update of EULAR recommendations for the treatment of systemic sclerosis. Ann Rheum Dis.

[133] Denton CP (2015). Systemic sclerosis: from pathogenesis to targeted therapy. Clin Exp Rheumatol.

[134] Khanna D, Furst DE, Hays RD, Park GS, Wong WK, Seibold JR, Mayes MD, White B, Wigley FF, Weisman M, Barr W, Moreland L, Medsger TA, Steen VD, Martin RW, Collier D, Weinstein A, Lally EV, Varga J, Weiner SR, Andrews B, Abeles M, Clements PJ (2006). Minimally important difference in diffuse systemic sclerosis: results from the D-penicillamine study. Ann Rheum Dis.

[135] Khanna D, Furst DE, Wong WK, Tsevat J, Clements PJ, Park GS, Postlethwaite AE, Ahmed M, Ginsburg S, Hays RD, Scleroderma Collagen Type 1 Study Group (2007). Reliability, validity, and minimally important differences of the SF-6D in systemic sclerosis. Qual Life Res.

[136] Khanna D, Tseng CH, Furst DE, Clements PJ, Elashoff R, Roth M, Elashoff D, Tashkin DP, for Scleroderma Lung Study Investigators (2009). Minimally important differences in the Mahler’s Transition Dyspnoea Index in a large randomized controlled trial—results from the Scleroderma Lung Study. Rheumatology (Oxford).

[137] Sekhon S, Pope J, Canadian Scleroderma Research Group, Baron M The minimally important difference in clinical practice for patient-centered outcomes including health assessment questionnaire, fatigue, pain, sleep, global visual analog scale, and SF-36 in scleroderma. J Rheumatol 2010, 37:591–598 10.3899/jrheum.090375 20080913

[138] Khanna D, Furst DE, Maranian P, Seibold JR, Impens A, Mayes MD, Clements PJ, Getzug T, Hays RD (2011). Minimally important differences of the UCLA Scleroderma Clinical Trial Consortium Gastrointestinal Tract Instrument. J Rheumatol.

[139] Lillie EO, Patay B, Diamant J, Issell B, Topol EJ, Schork NJ (2011). The n-of-1 clinical trial: the ultimate strategy for individualizing medicine?. Per Med.

[140] Schork NJ (2015). Personalized medicine: Time for one-person trials. Nature.

[141] Gardeux V, Achour I, Li J, Maienschein-Cline M, Li H, Pesce L, Parinandi G, Bahroos N, Winn R, Foster I, Garcia JG, Lussier YA (2014). ‘N-of-1-pathways’ unveils personal deregulated mechanisms from a single pair of RNA-Seq samples: towards precision medicine. J Am Med Inform Assoc.

[142] Wild CP (2005). Complementing the genome with an “exposome”: the outstanding challenge of environmental exposure measurement in molecular epidemiology. Cancer Epidemiol Biomark Prev.

[143] Kim Kyoung-Nam, Hong Yun-Chul (2017). The exposome and the future of epidemiology: a vision and prospect. Environmental Health and Toxicology.

[144] Posada de la Paz M, Philen RM, Borda AI (2001). Toxic oil syndrome: the perspective after 20 years. Epidemiol Rev.

